# CRISPR-AsCas12f1 couples out-of-protospacer DNA unwinding with exonuclease activity in the sequential target cleavage

**DOI:** 10.1093/nar/gkae989

**Published:** 2024-11-12

**Authors:** Xiaoxuan Song, Ziting Chen, Wenjun Sun, Hao Yang, Lijuan Guo, Yilin Zhao, Yanan Li, Zhiyun Ren, Jin Shi, Cong Liu, Peixiang Ma, Xingxu Huang, Quanjiang Ji, Bo Sun

**Affiliations:** School of Life Science and Technology, ShanghaiTech University, Shanghai 201210, China; School of Life Science and Technology, ShanghaiTech University, Shanghai 201210, China; ENT Institute and Department of Otorhinolaryngology, Eye & ENT Hospital, State Key Laboratory of Medical Neurobiology and MOE Frontiers Center for Brain Science, NHC Key Laboratory of Hearing Medicine, Institutes of Biomedical Sciences, Fudan University, Shanghai 200031, China; School of Life Science and Technology, ShanghaiTech University, Shanghai 201210, China; School of Life Sciences and Biotechnology, Shanghai Jiao Tong University, Shanghai 200240, China; School of Life Science and Technology, ShanghaiTech University, Shanghai 201210, China; School of Life Science and Technology, ShanghaiTech University, Shanghai 201210, China; School of Life Science and Technology, ShanghaiTech University, Shanghai 201210, China; School of Life Science and Technology, ShanghaiTech University, Shanghai 201210, China; CAS Center for Excellence in Molecular Cell Science, Shanghai Institute of Biochemistry and Cell Biology, Chinese Academy of Sciences, Shanghai 200031, China; University of Chinese Academy of Sciences, Beijing 100049, China; School of Physical Science and Technology, ShanghaiTech University, Shanghai 201210, China; Interdisciplinary Research Center on Biology and Chemistry, Shanghai Institute of Organic Chemistry, Chinese Academy of Sciences, Shanghai 201210, China; State Key Laboratory of Chemical Biology, Shanghai Institute of Organic Chemistry, Chinese Academy of Sciences, Shanghai 200032, China; Shanghai Key Laboratory of Orthopedic Implants, Department of Orthopedic Surgery, Shanghai Ninth People's Hospital, Shanghai Jiao Tong University School of Medicine, Shanghai200025, China; Zhejiang Lab, Hangzhou, Zhejiang 311121, China; School of Physical Science and Technology, ShanghaiTech University, Shanghai 201210, China; Gene Editing Center, ShanghaiTech University, Shanghai 201210, China; School of Life Science and Technology, ShanghaiTech University, Shanghai 201210, China; Gene Editing Center, ShanghaiTech University, Shanghai 201210, China

## Abstract

Type V-F CRISPR-Cas12f is a group of hypercompact RNA-guided nucleases that present a versatile *in vivo* delivery platform for gene therapy. Upon target recognition, *Acidibacillus sulfuroxidans* Cas12f (AsCas12f1) distinctively engenders three DNA break sites, two of which are located outside the protospacer. Combining ensemble and single-molecule approaches, we elucidate the molecular details underlying AsCas12f1-mediated DNA cleavages. We find that following the protospacer DNA unwinding and non-target strand (NTS) DNA nicking, AsCas12f1 surprisingly carries out bidirectional exonucleolytic cleavage from the nick. Subsequently, DNA unwinding is extended to the out-of-protospacer region, and AsCas12f1 gradually digests the unwound DNA beyond the protospacer. Eventually, the single endonucleolytic target-strand DNA cleavage at 3 nt downstream of the protospacer readily dissociates the ternary AsCas12f1-sgRNA–DNA complex from the protospacer adjacent motif-distal end, leaving a staggered double-strand DNA break. The coupling between the unwinding and cleavage of both protospacer and out-of-protospacer DNA is promoted by Mg^2+^. Kinetic analysis on the engineered AsCas12f1-v5.1 variant identifies the only accelerated step of the protospacer NTS DNA trimming within the sequential DNA cleavage. Our findings provide a dynamic view of AsCas12f1 catalyzing DNA unwinding-coupled nucleolytic cleavage and help with practical improvements of Cas12f-based genome editing tools.

## Graphical Abstract

**Graphical Abstract:**
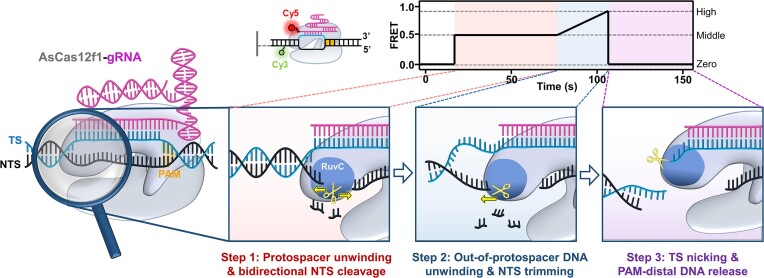

## Introduction

Clustered Regularly Interspaced Short Palindromic Repeats (CRISPR)-associated (Cas) systems function as an adaptive immune defense against foreign genetic elements in most bacteria and archaea (1–3). In class II CRISPR-Cas systems, single Cas effectors, such as Cas9 and Cas12a (1000∼1600 a.a.), specifically identify and cleave foreign DNA or RNA based on complementarity to a guide RNA (gRNA) (4–8). These well-characterized systems have been exploited as programmable genome tools that are widely used across various organisms (9–11). However, the *in vivo* delivery of these Cas effectors with engineered single-guided RNA (sgRNA) is often restricted by the cargo-size-limited adeno-associated virus (AAV) vector, limiting specific applications (12,13). Based on the metagenomics analysis of microbial communities, class II type V-F Cas12f effectors, such as Un1Cas12f1, SpaCas12f1, CnCas12f1, OsCas12f1 and RhCas12f1, have recently been identified as a group of exceptionally compact RNA-guided endonucleases (400∼700 a.a.) that recognize 5′ T-rich or C-rich protospacer adjacent motifs (PAMs) (14–19). AAV-mediated delivery of Cas12f proteins and their cognate sgRNAs has led to successful genome editing in bacteria, human cells and mice, underlining their therapeutic potential (19–22). Comprehensive characterization of Cas12f targeting and cleaving DNA is essential for uncovering their functional limitations and guiding rational protein and gRNA engineering for improved genome modifications.

Of these known Cas12f proteins, *Acidibacillus sulfuroxidans* Cas12f (AsCas12f1, 422 a.a.) possesses innate plasmid interference activity and has been developed as an active genome editor (15,19). Compared to the widely used SpCas9 and AsCas12a, AsCas12f1 presents a moderate level of indel activity (19,23). Additionally, the engineered AsCas12f1 systems, such as enAsCas12f1 and AsCas12f1-v5.1, have higher *in vitro* DNA cleavage activity and *in vivo* genome editing efficiency, holding promise for better performance as DNA editing tools (20,24,25). To comprehensively understand the AsCas12f1 nuclease, biochemical and structural studies have been extensively conducted to characterize the molecular details underlining its interplay with DNA target. Specifically, AsCas12f1 recognizes a 5′-NTTR PAM sequence and requires a specific 20-nt protospacer DNA sequence (15,19). Upon PAM recognition, AsCas12f1-gRNA displaces the non-target strand (NTS) DNA and initiates base pairing between the CRISPR RNA (crRNA) and the PAM-proximal bases of the target strand (TS) DNA (20,24,25). The extension of the RNA–DNA base pairing to the PAM-distal end allows the formation of the full-length heteroduplex structure, which licenses DNA degradation (25). Intriguingly, unlike Cas9 and Cas12a, which introduce single cleavage sites on both TS and NTS DNA within the protospacer region, AsCas12f1 is distinct in creating three asymmetrical DNA breaks (19). Among the three break sites, only one is within the protospacer region, located ∼12 nt downstream of the PAM on the NTS DNA (termed NTS-P); the other two are situated unexpectedly outside the protospacer DNA on both NTS (∼5 nt downstream of the protospacer, termed NTS-O) and TS (∼3 nt downstream of the protospacer, termed TS-O) DNA, resulting in two staggered double-strand breaks in DNA (19). Meanwhile, the cryoelectron microscopy (cryo-EM) studies have revealed asymmetrically dimerized AsCas12f1 proteins in association with sgRNA and target DNA (25). Although two RuvC domains exist in the AsCas12f1 dimer, only one is active and responsible for all DNA cleavage activity (25). Despite these valuable insights, the dynamic mechanism governing DNA target cleavage by a single RuvC domain of AsCas12f1 remains to be elucidated.

In this study, combining single-molecule and ensemble approaches, we systematically characterized the dynamic interaction of AsCas12f1 with DNA in real time and compared it with that of the engineered AsCas12f1-v5.1. These approaches enabled us to resolve the specific DNA binding, unwinding and cleavage steps of AsCas12f1 and quantify each step’s kinetics. To our surprise, AsCas12f1 exhibits bidirectional exonuclease activity on the NTS DNA and endonuclease activity on the TS DNA in a sequential manner. Significantly, AsCas12f1 has evolved a two-step DNA unwinding mechanism that primes the protospacer and out-of-protospacer DNA cleavage. Mg^2+^ plays critical regulatory roles in the tight coupling between DNA unwinding and cleavage. Compared with wild-type (WT) AsCas12f1, AsCas12f1-v5.1 displayed a faster rate of processing the protospacer NTS DNA, whereas their DNA targeting and out-of-protospacer DNA processing kinetics are comparable. On the basis of these findings, we propose a detailed model of AsCas12f1 coupling two-step DNA unwinding with both exonuclease and endonuclease activities for the DNA target cleavage.

## Materials and methods

### Expression and purification of AsCas12f1 proteins

AsCas12f1 was expressed and purified as follows. A pET28a-based expression vector containing a sequence encoding AsCas12f1 (AsCas12f1 residues 1 to 422) and an N-terminal SUMO-tag with His_6_-tag. The SUMO fusion protein tag promotes soluble expression of AsCas12f1. The proteins were expressed in *Escherichia coli* strain BL21 (DE3) (TransGen Biotech) cells that were grown in Luria-Bertani (LB) medium at 37°C for a few hours. When the optical density at 600 nm reached 0.6, protein expression was performed at 16°C for 16 h following the induction with 0.25 mM isopropyl β-D-thiogalactopyranoside. The medium was then discarded, and the cells were harvested. The harvested cells were lysed in 20 mM Tris-HCl (pH = 7.5), 1 M NaCl, 15 mM imidazole, 1 mM dithiothreitol (DTT) and 1% protease inhibitor and passed through a homogenizer three times at ∼1000 bar. The lysed dilution was then ultracentrifuged at 11 000 rpm for 1 h and the clarified supernatant from the cell lysate was separated from the cellular debris and bound in batch to Ni-NTA Agarose (TransGen Biotech). The resin was washed extensively with buffer containing 50 mM Tris-HCl (pH = 7.5), 1 M NaCl and 15 mM imidazole, and the bound protein was eluted by lysis buffer containing different imidazole concentrations (50, 100, 250 and 500 mM). The bulk of AsCas12f1 was in the 250 mM imidazole eluate. The eluate was then concentrated using 30-kDa filtration at 5000 rpm to a certain volume at 4°C. The eluted protein was buffer-exchanged by centrifugation to remove imidazole, and then HRV3C was added for 24-h incubation at 4°C to remove the SUMO tag. After that, AsCas12f1 was subject to gel filtration chromatography with a Superdex 200 10/30 column (GE Healthcare) in storage buffer [20 mM Tris-HCl (pH = 7.5), 1M NaCl, 1 mM DTT and 10% glycerol]. The proteins were stored at −80°C before use.

The dead version of AsCas12f1 (dAsCas12f1) and AsCas12f1-v5.1 were created as previously described (25), and both proteins were expressed and purified similarly.

### Preparation of DNA templates and gRNAs

DNA oligonucleotides with or without labels were purchased from Sangon Biotech (Shanghai, China) (Supplementary Table S1). NTS and TS DNA oligonucleotides at a molar ratio of 2:1 were mixed in buffer [20 mM Tris-HCl (pH = 8.0) and 50 mM NaCl], heated to 95°C for 3 min and then slowly cooled to room temperature over 2 h.

The pUC57-sgRNA and pUC57-tracrRNA (trans-activating CRISPR RNA) expression vectors were linearized by polymerase chain reaction amplification, and the sgRNA and tracrRNA were transcribed using the T7 High-Efficiency Transcription Kit (Transgen Biotech). The sgRNA and tracrRNA were then purified using an EasyPure^®^ RNA Purification Kit (Transgen Biotech). Cy5-labeled crRNA was purchased from GenScript (Nanjing, China). The sequences of the sgRNA, tracrRNA, crRNA and the primers used in this study are listed in Supplementary Table S1.

In the single-molecule DNA unzipping assay, the DNA template consisting of two arms and a trunk was prepared as previously described (Supplementary Figure S1) (26).

### *In vitro* DNA cleavage assay

For the bulk DNA cleavage assay, AsCas12f1 was first complexed with sgRNA at a 1:2 ratio at 45°C or 37°C for 5 min in reaction buffer [20 mM Tris-HCl (pH = 7.5), 50 mM NaCl and 1–10 mM MgCl_2_]. The complexed AsCas12f1 (500 nM) was incubated with the annealed DNA substrate (5 nM) for the indicated time at 45°C or 37°C before quenching with formamide gel loading buffer supplemented with 50 mM ethylenediaminetetraacetic acid (EDTA), followed by heating at 95°C for 10 min. The reaction products were resolved by 12% denaturing polyacrylamide gel electrophoresis (7 M urea PAGE) in TBE and visualized using a Typhoon FLA 9500 (GE Healthcare). Each experiment was repeated in triplicate.

### Single-molecule fluorescence resonance energy transfer (FRET) experiments

Single-molecule FRET (smFRET) experiments were performed using total internal reflection fluorescence (TIRF) microscopy. Quartz slides and coverslips (Fisher Scientific, USA) were coated with polyethylene glycol (PEG) to minimize surface interactions with the DNA. Briefly, the slides and coverslips were cleaned and treated with methanol, acetone and potassium hydroxide. The coverslip surfaces were then treated with aminosilane and coated with a mixture of 99% mPEG-5000 (Laysan Bio, Inc.) and 1% biotin-PEG-5000 (Laysan Bio, Inc.).

DNA molecules were annealed and immobilized on the PEG-passivated surface via biotin-streptavidin interaction. Streptavidin (10 μg/ml) was added to the microfluidic chamber made of the PEG-coated coverslip and incubated for 5 min. After washing, DNA substrates (10–50 pM) were added to the chamber.

All smFRET measurements were performed at 45°C under the following buffer conditions unless stated otherwise: 20 mM Tris-HCl (pH = 7.5), 50 mM NaCl, varying concentrations of MgCl_2_ and an oxygen scavenging system (0.8% D-glucose, 1 mg/ml glucose oxidase, 0.4 mg/ml catalase and 1 mM Trolox) (27,28). The fluorescently labeled DNA substrate was immobilized on the glass surface. Then, a pre-assembled AsCas12f1-gRNA complex (15 nM) was injected into the chamber prior to data acquisition. The imaging was collected using two-color TIRF microscopy. An EMCCD camera (Andor) was used to record videos for all experiments with an exposure time of 100 ms for over 1500 frames. Each frame was further processed to extract single-molecule fluorescence intensities. Only fluorescence spots in the acceptor channel were used for analysis to avoid missing or inactivating the acceptors. Each experiment was performed at least three times to ensure reproducibility.

### smFRET data analysis

The FRET efficiency of a single molecule was approximated as *E*= I_A_/(I_D_+ I_A_), where I_D_ and I_A_ are the backgrounds and leakage-corrected emission intensities of the donor and acceptor, respectively (29,30). Single-molecule trajectories were analyzed using smCamera and exported at 10 Hz. Representative real-time trajectories were smoothed using an adjacent-averaging algorithm with *n* = 5 points. FRET contour plots were built from at least 60 selected trajectories that did not undergo fluorescence quenching using SPARTAN 3.7.0 (31). Dynamic FRET trajectories were analyzed using a Hidden Markov model (HMM)-based software HaMMy 4.0 (32). Transition density plots (TDPs) were generated using a custom code written in MATLAB. The fitting results were used to determine the dwell time in each state.

### Single-molecule DNA unzipping assay

An M-trap optical tweezer microscope from LUMICKS (Amsterdam, Netherlands) was used to perform the single-molecule optical tweezer assays. The sample chamber preparation was similar to that previously described (26,33). Briefly, glass coverslips were cleaned and functionalized with partially biotinylated PEG (Laysan Bio, Inc.), followed by coating with streptavidin (Thermo Fisher Scientific). DNA tethers were then formed by incubation with biotin-labeled DNA (Supplementary Figure S1). Antidigoxigenin-coated polystyrene microspheres (0.5 μm in diameter, Polysciences) were added to the chamber for the tether assembly.

AsCas12f1-sgRNA was incubated with the trunk DNA template in a 50:1 ratio in DNA unzipping buffer [20 mM Tris-HCl (pH = 7.5), 100 mM NaCl and 10 mM MgCl_2_]. This mixture was then diluted to the final experimental concentration and added to the chamber. Each experiment was conducted by mechanically unzipping the double-stranded DNA (dsDNA) at a slow velocity of 50 nm/s to probe potential interactions at the fork. The acquired data were converted into force in pN and DNA extension in nm as previously described (34). In the unzipping experiments, one separated base pair generated two nucleotides of single-stranded DNA (ssDNA). Accordingly, the real-time DNA extension in nanometer was converted into the number of base pairs unwound based on the elastic parameters of ssDNA under our experimental conditions (35). To improve precision and accuracy, the curves showing the force versus the number of base pairs unzipped were aligned to the theoretical curve by the cross-correlation of a region before and after the ternary complex disruption (34). At least 15 DNA molecules were examined in each experimental condition.

### Statistical analysis

Statistical significance was determined by unpaired two-tailed Student’s *t*-test using Origin. The difference between the two groups was considered statistically significant when the *P*-value was <0.05 (*P < 0.05; **P < 0.01; ***P < 0.001; ns, no significance).

## Results

### Bidirectional exonuclease activity in the sequential DNA cleavage of AsCas12f1

**Figure 1.**
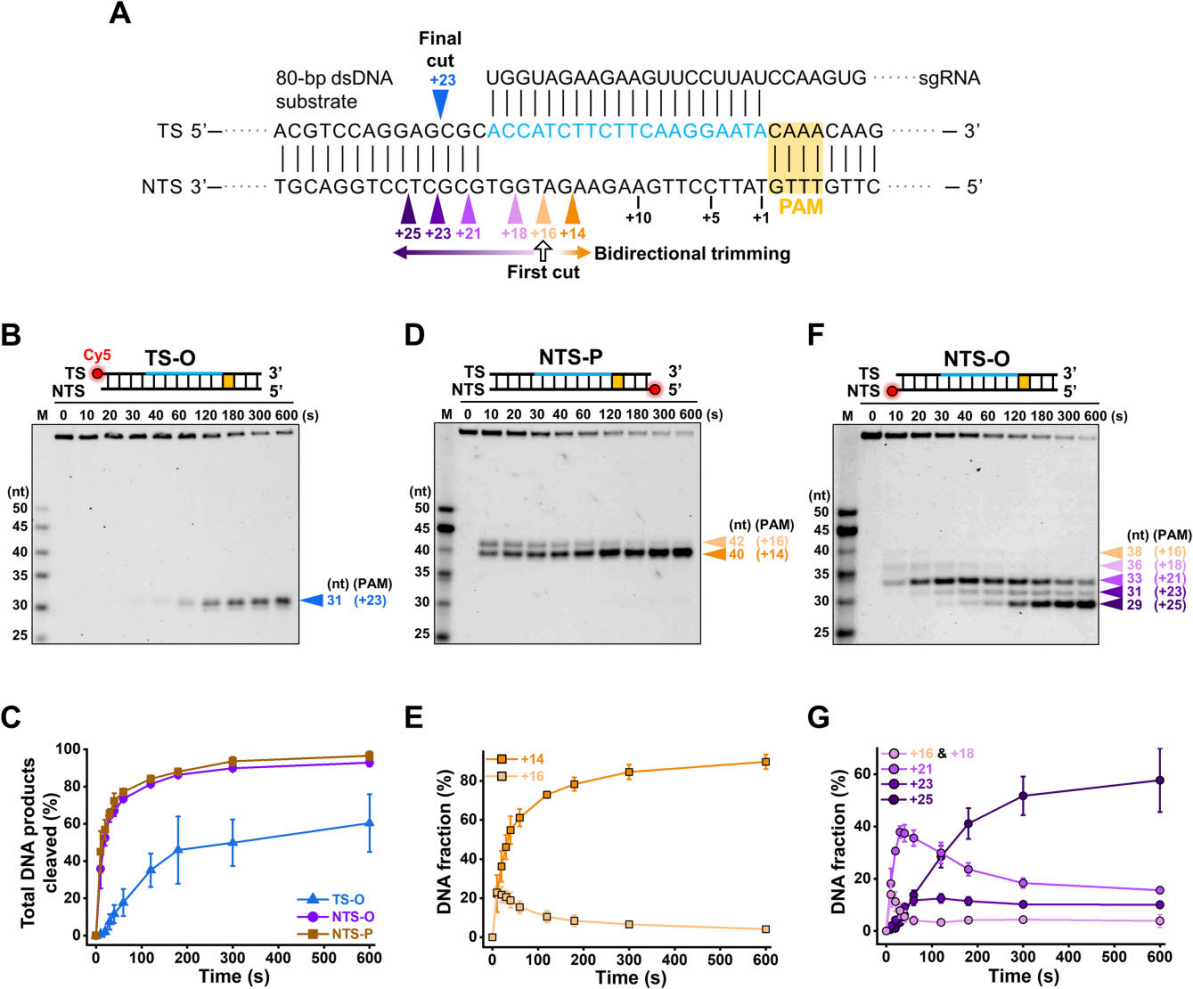
AsCas12f1 cleaves target DNA with both endonuclease and exonuclease activities. (**A**) A map for the dsDNA cleavage pattern of AsCas12f1. The protospacer DNA and the PAM are colored cyan and yellow, respectively. Colored triangles indicate the cleavage sites and directionality. (**B**) A representative gel of AsCas12f1-catalyzed DNA cleavage toward the TS-O substrate. (**C**) The total DNA substrates cleaved by AsCas12f1 as a function of time. (**D**) A representative gel of AsCas12f1-catalyzed DNA cleavage toward the NTS-P substrate. (**E**) The quantification of DNA cleavage products (+14 and +16) from panel (D) as a function of time. (**F**) A representative gel of AsCas12f1-catalyzed DNA cleavage toward the NTS-O substrate. (**G**) The quantification of DNA cleavage products (+16 and +18, +21, +23 and +25) from panel (F) as a function of time. Error bars in panels (C), (E) and (G) represent the standard deviation (S.D.) of three replicates.

We first aimed to address whether AsCas12f1 catalyzes DNA target cleavage in a specific order by examining the kinetics of the NTS and TS DNA cleavage. To this end, we prepared four identical 80-bp dsDNA substrates with a single Cy5 labeled at four different ends and a cognate sgRNA whose complementary DNA target is located between +1 (PAM-proximal side) and +20 (PAM-distal side) position (Figure 1A). Briefly, the AsCas12f1-sgRNA ribonucleoprotein (RNP) complex (500 nM) was incubated with Cy5-labeled DNA substrates (5 nM) for indicated periods. To better distinguish the intermediate cleavage products, we retarded the cleavage rates of AsCas12f1 by performing the reaction under 1 mM Mg^2+^ at 37°C instead of the optimal condition (10 mM Mg^2+^ and 45°C) (19). The reaction was then quenched with 50 mM EDTA and denatured by heating at 95°C for 10 min and the cleavage products were resolved by urea PAGE.

We started the cleavage experiments with the TS-O substrate, wherein a Cy5 dye was labeled at the 5′ end of the TS DNA (Figure 1B). A single 31-mer DNA product was immediately discerned on the PAGE gel after 30 s, and it accumulated along with the diminished intact substrates (Figure 1B and C and Supplementary Figure S2A). Labeling the Cy5 dye on the other end of the TS DNA produced a single 49-mer DNA cleavage product (Supplementary Figure S2B). These findings suggest a single TS DNA cleavage site at the +23 position conducted by AsCas12f1, consistent with a previous study (19).

Next, we examined the NTS DNA cleavage by labeling a Cy5 dye at the 5′ or 3′ end of the NTS (NTS-P and NTS-O substrates, respectively). In addition to the expected 40-mer DNA product corresponding to the NTS-P cleavage site at the +14 position, a 42-mer cleavage product was also observed with the NTS-P substrate (Figure 1D and Supplementary Figure S2C). Notably, the 42-mer product appearing at the first time point (10 s) diminished along with the accumulation of the 40-mer product (Figure 1E). These findings suggest that after the initial breakage at the +16 position on the NTS, AsCas12f1 further trimmed two nucleotides off the 3′ end, resulting in the NTS-P cleavage site (Figure 1A). Strikingly, experiments with the NTS-O substrate generated up to five different cleavage products from +16 position to +25 position with incisions separated by 2–3 nt (Figure 1F). The cleavage sites were precisely determined by quantifying the lengths of these cleavage products in a high-resolution gel (Supplementary Figure S2D). In detail, the faintly observed 38-mer and 36-mer products corresponding to the cleavage at the +16 and +18 positions quickly disappeared; the 33-mer and 31-mer products corresponding to the cleavage at the +21 and +23 positions initially accumulated and faded with time; the 29-mer product corresponding to the cleavage at the +25 position appeared the latest and continuously accumulated with time (Figure 1G). In addition, the cleavage pattern was also observed in the cleavage reaction initiated by adding Mg^2+^ to the pre-assembled AsCas12f1-sgRNA–DNA complex (Supplementary Figure S3). Experiments with an internally labeled substrate revealed an intermediate DNA product of <8 nt (Supplementary Figure S4A). Therefore, in addition to the 3′–5′ trimming, AsCas12f1 also executed the 5′-3′ exonucleolytic degradation from the +16 to the +25 position (Figure 1A). A similar cleavage pattern was detected in the experiments with another set of DNA substrates, albeit with varied cleavage sites (Supplementary Figure S5). Moreover, we observed comparable intermediate DNA products under the optimal reaction condition and confirmed a single AsCas12f1 nuclease executing the bidirectional NTS cleavage (Supplementary Figures S4B and S6). These findings suggest the inherent bidirectional exonuclease activity of AsCas12f1 rather than the stringent condition or the specific DNA substrate used.

From the kinetic perspective, both NTS-P and NTS-O substrates started to disappear at earlier time points with faster rates compared with the TS-O substrate (Figure 1B, D and F, and Supplementary Figure S7). Consistently, the intermediate cleavage products of the NTS DNA appeared earlier than that of the TS DNA (Figure 1B, D and F). Thereby, it is conceivable that the NTS DNA cleavage precedes the TS DNA. This notion is further supported by the finding that the kinetics of the latest NTS DNA cleavage site (+25) is comparable to that of the single cleavage on the TS DNA (+23) (Figure 1C and G).

Collectively, AsCas12f1 cleaves the DNA target in a sequential order. To be precise, it realizes the double NTS cleavage by nicking at the +16 position and subsequent bidirectional exonucleolytic trimming to the +14 and +25, respectively; the completion of the NTS DNA processing is closely followed by the final endonucleolytic TS cleavage at the +23 position (Figure 1A).

### Out-of-protospacer DNA unwinding and PAM-distal DNA release of AsCas12f1

**Figure 2.**
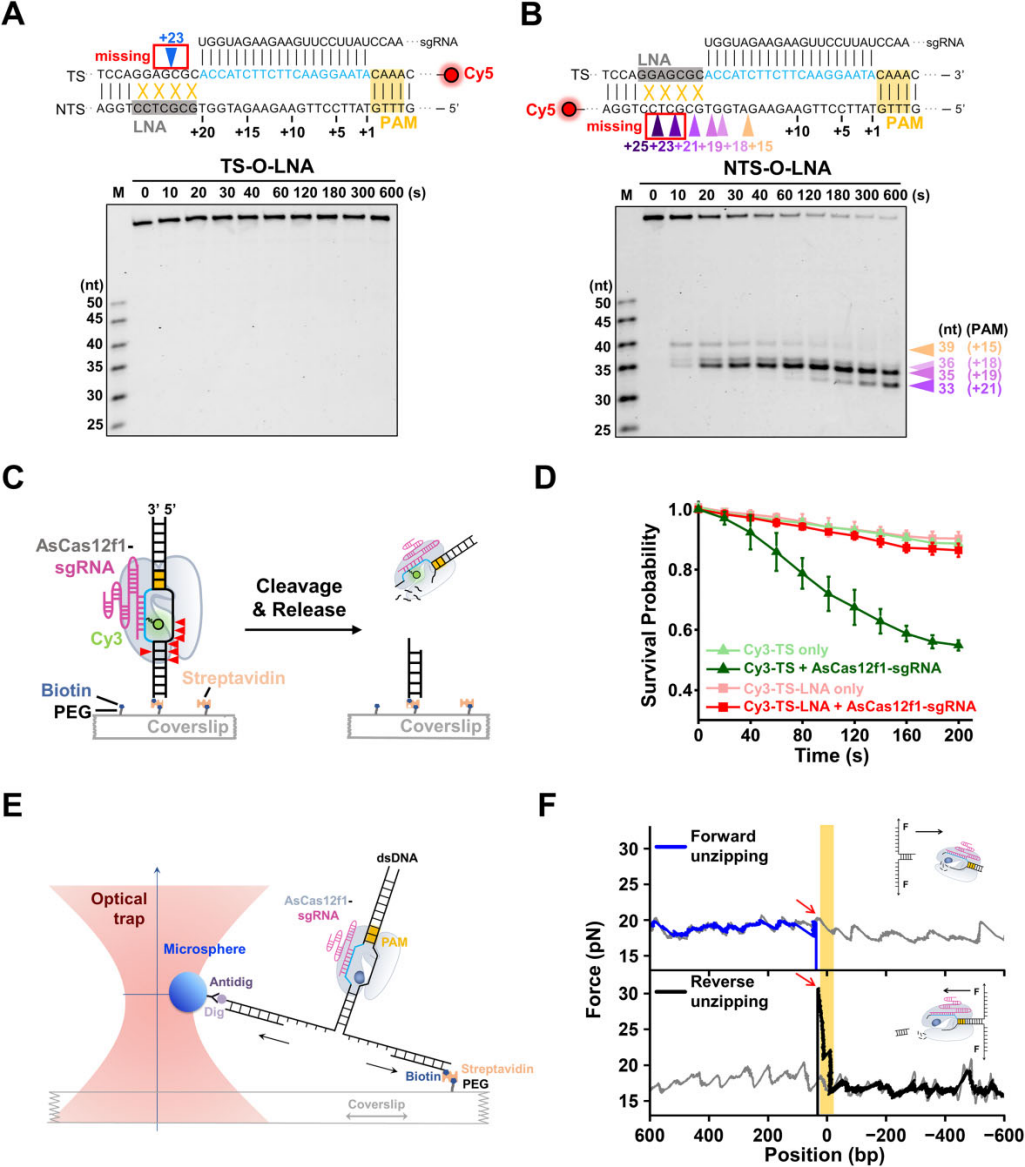
Out-of-protospacer DNA unwinding and PAM-distal DNA release by AsCas12f1. (**A,B**) The sequence pattern diagrams are shown above the representative gels of AsCas12f1-catalyzed DNA cleavage toward the Cy5-labeled TS-O-LNA (**A**) and NTS-O-LNA substrates (**B**). The protospacer DNA and the PAM are colored cyan and yellow, respectively. Colored triangles indicate the cleavage sites and those framed suggest they are missing. (**C**) Schematic diagram of the single-molecule experiment to monitor the release of the PAM-distal DNA after the DSB induced by AsCas12f1. (**D**) Survival probability of dwell times of Cy3 fluorescence on Cy3-TS (*n* = 262) or Cy3-TS-LNA (*n* = 144). Each data point is the average of three experiments. Error bars represent S.D. (**E**) A cartoon illustrating the single-molecule DNA unzipping experiment used to detect the interactions between AsCas12f1-gRNA and DNA. (**F**) Representative forward (blue) and reverse (black) DNA unzipping signatures in the presence and absence (gray) of AsCas12f1. The yellow rectangle shows the expected AsCas12f1 binding position. Red arrows indicate the tether breakage.

A prerequisite for Cas effectors to execute DNA cleavage is the unwinding of the target dsDNA to generate ssDNA (36–38). Our ensemble studies identified four cleavage sites of AsCas12f1 that are located outside the protospacer (Figure 1). We, therefore, asked whether out-of-protospacer DNA is also unwound by AsCas12f1 prior to these cleavage activities. To examine that, we prepared two Cy5-labeled DNA substrates with locked nucleic acid (LNA) modifications on the out-of-protospacer DNA region at positions +21 to +27 (Figure 2A and B). These nucleotide modifications are acknowledged to make the out-of-protospacer region less easily unwound (39,40) and yet interfere with nuclease-catalyzed DNA cleavage (41,42). Thus, we modified the out-of-protospacer NTS DNA to perturb out-of-protospacer DNA unwinding (termed TS-O-LNA substrate) and examined whether the single endonucleolytic cleavage on the unmodified TS DNA is allowed. The unnoticeable DNA cleavage product proved that AsCas12f1-catalyzed TS DNA cleavage was indeed prohibited on this substrate even in the optimal cleavage condition (Figure 2A and Supplementary Figure S8A). We then modified the opposite TS nucleotides (termed NTS-O-LNA substrate) and examined the NTS DNA cleavage by AsCas12f1. Along with the diminished 39-mer (+15 position) and 36-mer (+18 position) DNA products, 35-mer (+19 position) and 33-mer (+21 position) DNA products were accumulating (Figure 2B). Notably, compared with the unmodified NTS-O substrate, the farthest two NTS cleavage products (+23 and +25 positions) were missing with the NTS-O-LNA substrate (Figure 2B). In the optimal cleavage condition, the NTS nucleotides at positions +23 to +25 remain intact as well (Supplementary Figure S8B). The absence of the two farthest NTS DNA cleavage products and the TS DNA cleavage product supports that the LNA-modified DNA cleavage by AsCas12f1 is restrained within the protospacer region. Therefore, out-of-protospacer DNA unwinding is a prerequisite for AsCas12f1 to cleave the corresponding positions. In support of this notion, AsCas12f1 resumed the ability to trim off out-of-protospacer nucleotides on the NTS once they were not paired with the LNA-modified TS strand (Supplementary Figure S9). Additionally, pre-separation of the DNA region outside the protospacer led to multiple cleavage sites on the NTS beyond the protospacer. (Supplementary Figure S10).

After the DNA cleavage, the AsCas12f1-sgRNA complex may actively dissociate from the DNA target. We used a TIRF-based fluorescence approach to quantitatively investigate the DNA release by the RNP complex. To this aim, we prepared a DNA substrate with a Cy3 dye on the +16 nucleotide of the TS as a probe for DNA dissociation (Cy3-TS) and a biotin on the 5′ end of the same strand for surface immobilization. As shown in Figure 2C, the final TS cleavage by AsCas12f1 may cause the detachment of the Cy3-labeled PAM-proximal DNA from the surface-immobilized PAM-distal DNA due to the double-strand DNA break. After introducing the pre-assembled RNP complex, we recorded the number of fluorescently labeled DNA substrates. Indeed, the RNP complex actively cleaved approximately half of the fluorescently labeled DNA substrate within 200 s, favoring the target DNA cleavage by AsCas12f1 and the release of the PAM-distal DNA (Figure 2D and Supplementary Figure S11). In stark contrast, the LNA modifications on the out-of-protospacer DNA (Cy3-TS-LNA) significantly retained the fluorescence signal in 200 s (Figure 2D and Supplementary Figure S11). The distinct outcomes also support the incomplete TS DNA cleavage of the LNA-modified DNA substrate by AsCas12f1.

To further validate the PAM-distal DNA release by AsCas12f1, we employed an optical tweezers-based DNA unzipping assay that allows to mechanically separate two strands of AsCas12f1-cleaved dsDNA from the downstream or upstream side of the PAM (termed forward and reverse DNA unzipping, respectively) (Figure 2F and Supplementary Figure S1) (43–46). In the forward DNA unzipping assay, we measured the tether breakage close to the expected RNP binding position, indicating the DNA cleavage and PAM-distal DNA release by the complex (Figure 2F). However, the reverse DNA unzipping assay gave rise to a sudden rise above those of the naked DNA baseline at the expected RNP binding position prior to the tether breakage, suggesting the maintenance of the association of the RNP complex with the PAM-proximal DNA after the cleavage (Figure 2F). The naked PAM-distal DNA and the RNP-associated PAM-proximal DNA after the cleavage confirmed the quick release of the PAM-distal DNA by the AsCas12f1-sgRNA complex.

Altogether, AsCas12f1 requires the separation of the out-of-protospacer DNA region for the cleavage on the corresponding DNA regions, and the final TS DNA cleavage readily dissociates the RNP complex from the PAM-distal end, leaving a staggered double-strand DNA break.

### Two discrete DNA unwinding steps prime the sequential target cleavage of AsCas12f1

**Figure 3.**
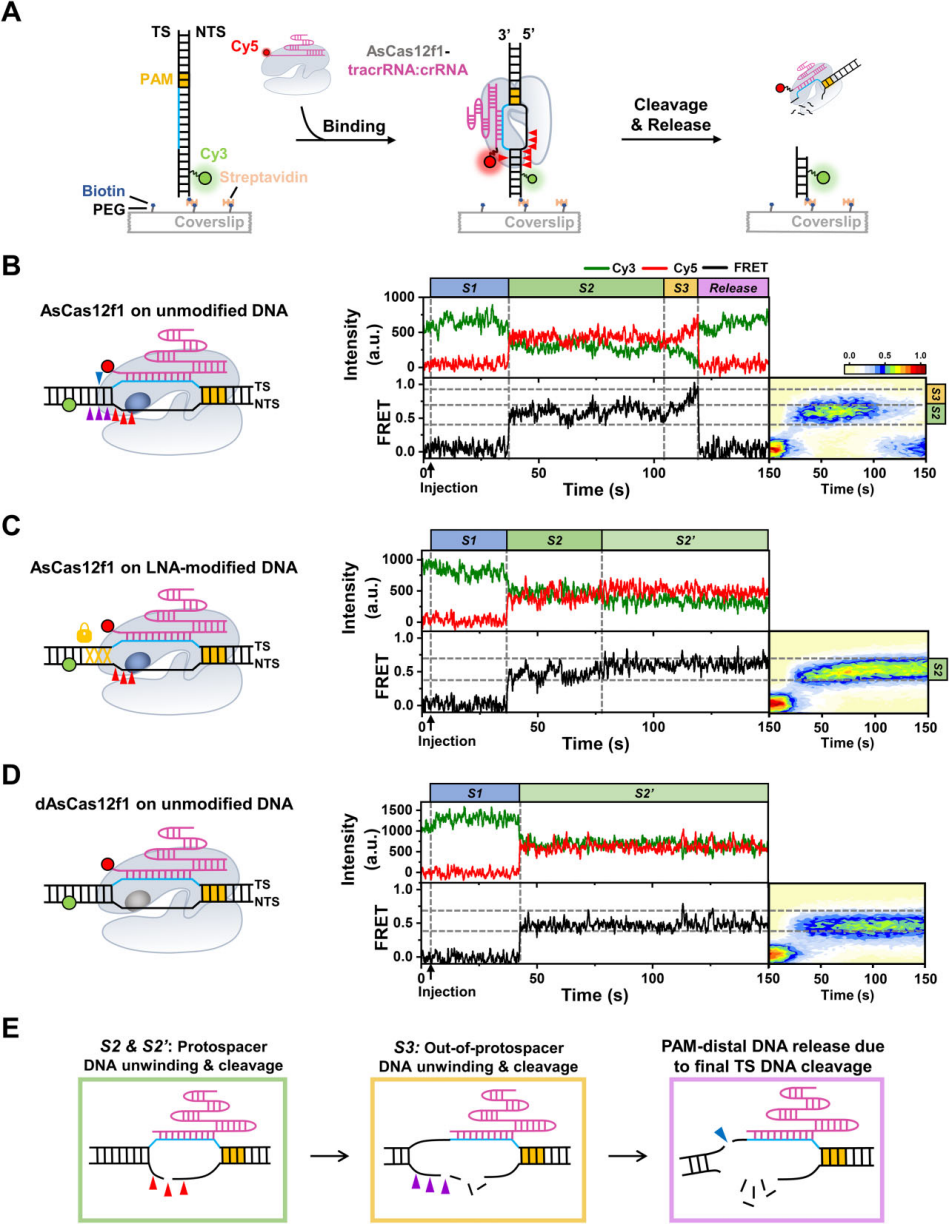
AsCas12f1-mediated two-step DNA unwinding and cleavage detected by smFRET. (**A**) A schematic diagram of the smFRET experiment to monitor the binding and cleavage reaction of different DNA substrates by AsCas12f1 and dAsCas12f1. (**B**) A cartoon illustrating the smFRET experiment to detect DNA binding and cleavage by AsCas12f1. A representative FRET trajectory is shown on the right. FRET contour plots were built from 63 trajectories. (**C**) A cartoon illustrating the smFRET experiment to detect the binding and cleavage of AsCas12f1 toward the LNA-modified DNA substrate. A representative FRET trajectory is shown on the right. FRET contour plots were built from 115 trajectories. (**D**) A cartoon illustrating the smFRET experiment to detect DNA binding by dAsCas12f1. A representative FRET trajectory is shown on the right. FRET contour plots were built from 106 trajectories. Colored triangles indicate the cleavage sites at different locations. The active and inactive RuvC domains are highlighted in dark blue and gray, respectively. FRET contour plots are shown on the right of the real-time trajectories. (**E**) Cartoons illustrating the three DNA states detected in the smFRET assay.

Our findings raised a question of how AsCas12f1 coordinates the unwinding of protospacer and out-of-protospacer DNA in the sequential DNA target cleavage. To address this question, we set out to perform smFRET experiments to monitor the dynamics of AsCas12f1-catalyzed DNA cleavage in real time. In this assay, we labeled a donor (Cy3) at the +34 position on the NTS DNA and an acceptor (Cy5) at the 3′ end of the crRNA (Figure 3A). Additionally, the biotin on the 3′ end of the NTS DNA allowed for the immobilization of the substrate on a streptavidin-coated quartz surface. The hybridization between crRNA and protospacer DNA would spatially bring the acceptor close to the donor and thus cause an increase in FRET efficiency (*E*) (Figure 3A). As expected, upon addition of the AsCas12f1-tracrRNA-crRNA complex to the immobilized DNA substrate, 45% of real-time FRET trajectories (*n* = 119) showed that *E* starting around 0 (termed *S1* state) drastically increased to ∼0.5, which ended up with the sudden drop to 0 again due to the loss of the acceptor signal (Figure 3B). The merely detected rebinding of AsCas12f1-gRNA to these post-reactive substrates (31 out of 598 substrates) suggests that the disappearance of the Cy5 signal is due to the RNP complex cleaving the target and releasing the PAM-distal DNA. Using a partially matched dsDNA, we provided evidence that the *E* value of ∼0.5 is a result of the full-length hybridization between the crRNA and the DNA target (Supplementary Figure S12). Interestingly, following the initial binding of the RNP complex to the DNA substrate, *E* values slowly fluctuated between 0.4 and 0.7 and then rose to ∼0.9 right before the PAM-distal DNA release, which hereafter are designated the *S2* and *S3* states, respectively (Figure 3B). To determine the origin of these specific FRET states, we revisited the smFRET experiments with the LNA-modified DNA substrate to block the unwinding of out-of-protospacer DNA. With this DNA substrate, the *S3* state and the loss of the acceptor signal were missing, whereas the *S1* and *S2* states still existed. The fluctuated *S2* state eventually stabilized around 0.6 (termed *S2*′ state) (Figure 3C). Therefore, the *S3* state is attributable to the out-of-protospacer DNA unwinding and cleavage by AsCas12f1. Since AsCas12f1 can still perform the protospacer DNA cleavage on the LNA-modified DNA (Figure 2B), the *S2* and *S2*′ states possibly reflect the catalytic reactions on the protospacer DNA. To further clarify these two FRET states, we assayed with a dead version of the AsCas12f1 nuclease (dAsCas12f1) (25). It was found that after the dAsCas12f1–tracrRNA–crRNA complex bound the DNA substrate, *E* directly entered the stable *S2*′ state, and the fluctuated *S2* state was not detectable (Figure 3D). Given that dAsCas12f1 is incapable of cleaving DNA, the *S2*′ state presumably reflects the stable R-loop formation without DNA cleavage, and the fluctuation of *E* in the *S2* state likely originates from the bidirectional trimming of the protospacer NTS DNA.

Collectively, our smFRET results strongly support a two-step DNA unwinding and cleavage mechanism of AsCas12f1: the RNP complex first unwinds and cleaves the protospacer DNA; following that, additional out-of-protospacer DNA unwinding occurs, which allows AsCas12f1 to cleave the NTS and TS DNA beyond the protospacer (Figure 3E).

### Mg^2+^ promotes the coupling between DNA unwinding and cleavage of AsCas12f1

**Figure 4.**
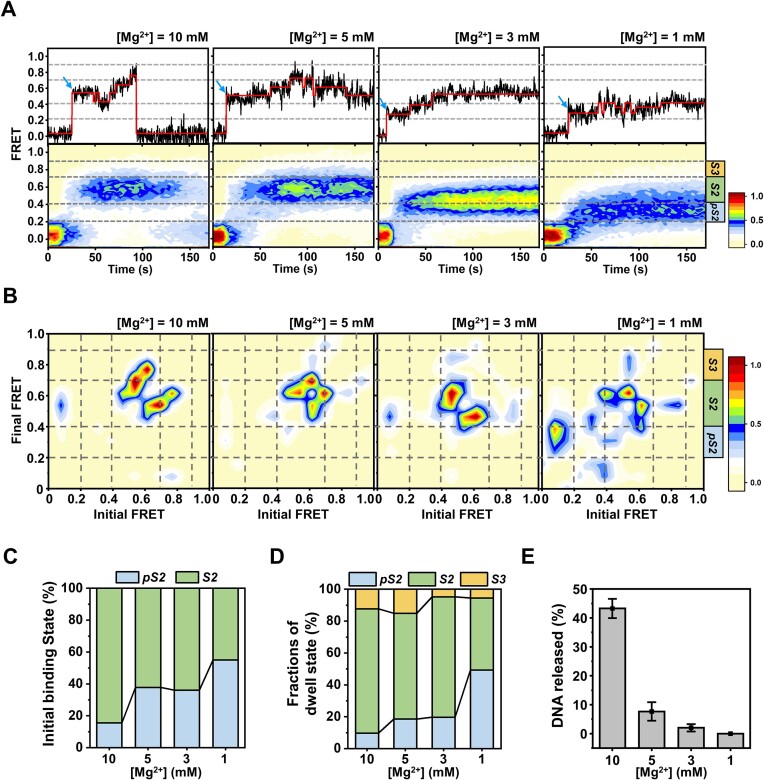
Effects of Mg^2+^ on AsCas12f1-catalyzed DNA unwinding and cleavage. (**A**) Representative FRET trajectories (top) and corresponding FRET contour plots (bottom) showing the dynamics of the AsCas12f1-gRNA complex interacting with DNA substrate under 10 mM Mg^2+^ (*n* = 124), 5 mM Mg^2+^ (*n* = 100), 3 mM Mg^2+^ (*n* = 137) and 1 mM Mg^2+^ (*n* = 104) concentrations. Blue arrows indicate the initial *E* state after the AsCas12f1 binding to the DNA substrate. (**B**) TDPs from HMM analysis of the FRET trajectories under 10 mM Mg^2+^ (*n* = 124), 5 mM Mg^2+^ (*n* = 100), 3 mM Mg^2+^ (*n* = 137) and 1 mM Mg^2+^ (*n* = 104) concentrations. In the TDPs, the FRET values before and after each transition were plotted as a normalized heat map. Gray dashed lines illustrate the transitions in three distinct reaction states. (**C**) Fractions of the initial *E* states resulting from the AsCas12f1 binding to the target DNA under 10 mM Mg^2+^ (*n* = 124), 5 mM Mg^2+^ (*n* = 100), 3 mM Mg^2+^ (*n* = 137) and 1 mM Mg^2+^ (*n* = 104) concentrations. (**D**) Fractions of different *E* states after AsCas12f1 binding to the target DNA under 10 mM Mg^2+^ (*n* = 124), 5 mM Mg^2+^ (*n* = 100), 3 mM Mg^2+^ (*n* = 137) and 1 mM Mg^2+^ (*n* = 104) concentrations. (**E**) Fractions of DNA trajectories that show the PAM-distal DNA release under 10 mM Mg^2+^ (*n* = 124), 5 mM Mg^2+^ (*n* = 100), 3 mM Mg^2+^ (*n* = 137) and 1 mM Mg^2+^ (*n* = 104) concentrations. Error bars represent the S.D. of three replicates.

Our gel-based results agree with a previous study that the DNA cleavage activity of AsCas12f1 is stringently regulated by Mg^2+^ (Supplementary Figure S13) (40). Since we have resolved the detailed dynamics of AsCas12f1-mediated DNA unwinding and cleavage, we sought to identify the regulatory role of Mg^2+^ in this dynamic process. Statistical comparisons of the dwell times on the *S1* state (*t_1_*) have proved that the effect of Mg^2+^ on the association of AsCas12f1 to the target DNA is negligible (Supplementary Figure S14). We then repeated the smFRET experiment to examine the interaction between AsCas12f1-gRNA and the target DNA under varied Mg^2+^ concentrations. With the decrease in Mg^2+^ concentration from 10 mM to 1 mM, real-time FRET trajectories exhibited distinct features across a time window of 170 s (Figure 4A). We performed the HMM on these trajectories and obtained the TDPs to characterize and compare these features (Figure 4A and B).

First, lowering the Mg^2+^ concentration caused the increase in the fraction of a low *E* state between 0.2 and 0.4 after the initial complex binding (Figure 4A–C). According to the control experiment with a partially matched sgRNA, this low *E* state is possibly a premature *S2* state (hereafter termed *pS2* state) resulting from the partial crRNA–DNA hybridization (Supplementary Figure S12). Statistical analysis showed that the *pS2* state dominates over the *S2* state under low Mg^2+^ concentrations (Figure 4C). Second, even though *E* mainly dwelled on the *S2* state after the initial binding, reducing Mg^2+^ concentration led to the less visited *S3* state and, instead, the elevated fraction of the *pS2* state (Figure 4B and D). These findings can be reasoned by AsCas12f1 being sequestered in dealing with the protospacer DNA and leaving out-of-protospacer DNA untouched. Third, consistent with the less visited *S3* state, the TS DNA cleavage and the PAM-distal DNA release by AsCas12f1 are significantly attenuated in 170 s with the decrease in Mg^2+^ concentration (Figure 4E). Notably, despite the moderately elevated population of the *S3* state, a significant amount of the TS DNA was not cleaved by AsCas12f1 under 5 mM Mg^2+^ (Figure 4D and E). In this experimental condition, the *S3* state often switched back to the *S2* state instead, suggesting the occurrence of out-of-protospacer DNA rewinding (Figure 4A and B).

Our findings unveiled the regulatory role of Mg^2+^ in AsCas12f1 catalyzing DNA unwinding and cleavage. In conclusion, a high amount of Mg^2+^ ensures the formation of the full-length RNA–DNA heteroduplex structure and the coupling between the out-of-protospacer DNA unwinding and cleavage.

### AsCas12f1-v5.1 accelerates the kinetics of the protospacer DNA cleavage only

**Figure 5.**
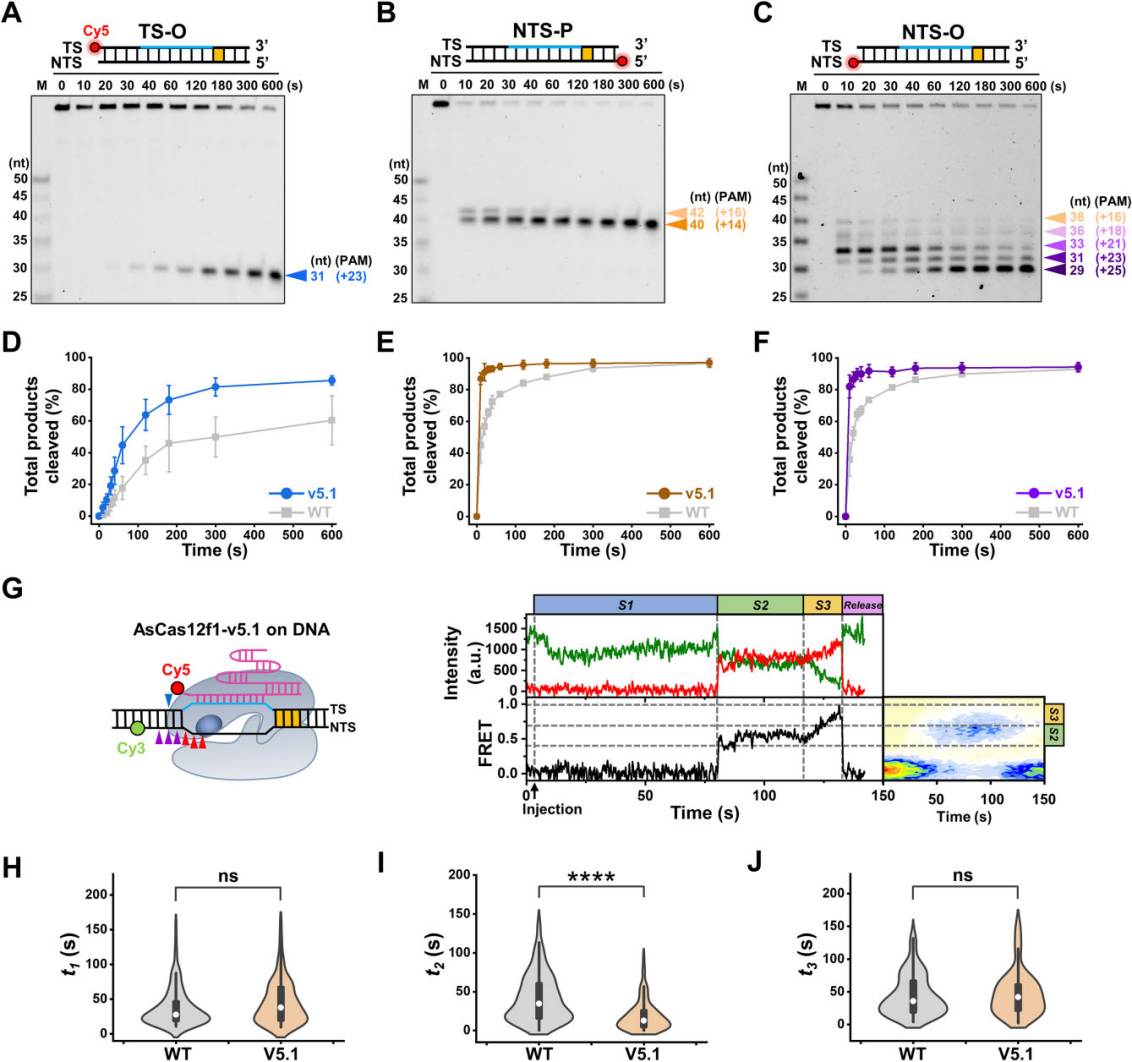
Kinetic characterization of AsCas12f1-v5.1 and comparison with WT AsCas12f1. (**A–C**) Representative gels of AsCas12f1-v5.1-catalyzed DNA cleavage toward the TS-O (**A**), NTS-P (**B**) and NTS-O substrates (**C**). Colored triangles indicate the cleavage sites. (**D–F**) The TS-O (**D**), NTS-P (**E**), and NTS-O (**F**) substrates cleaved by AsCas12f1-v5.1 and WT AsCas12f1 as a function of time. Error bars represent S.D. of three replicates. (**G**) A cartoon illustrating the smFRET experiment to detect DNA binding and cleavage by AsCas12f1-v5.1. The active RuvC domain is highlighted in dark blue. A representative FRET trajectory is shown on the right. Colored triangles indicate the cleavage sites at different locations. FRET contour plots are shown on the right of the real-time trajectory. FRET contour plots were built from 66 trajectories. The gray dashed lines are plotted to clarify different *E* states. (**H–J**) Statistical comparisons of the dwell times of *t_1_* (**H**), *t_2_* (**I**) and *t_3_* (**J**) on the respective *S1*, *S2* and *S3* states between AsCas12f1-v5.1 (*n* = 96) and WT AsCas12f1 (*n* = 122). In the violin plots, white dots represent the mean and black bars show the interquartile range (IQR) (thick bars) and 1.5 times IQR (thin bars). ns, no significance, ****P < 0.0001.

According to a recent study, an engineered AsCas12f1 variant, AsCas12f1-v5.1, displayed significantly enhanced insertion and deletion activities in human cells (25). To better understand how these improvements are made at the molecular level, we prepared AsCas12f1-v5.1 and compared it with WT AsCas12f1. We first performed the ensemble DNA cleavage assays with the three Cy5-labeled DNA substrates and found that AsCas12f1-v5.1 also cleaved the NTS DNA prior to the TS DNA (Figure 5A–F). Notably, the time-sensitive, ladder-like DNA cleavage patterns resembling those with the wide-type AsCas12f1 were also observable on the NTS-O and NTS-P substrates, indicating that the bidirectional exonuclease activity is conserved with AsCas12f1-v5.1 (Figure 5B and C and Supplementary Figure S15). In addition, in agreement with the *in vivo* data, AsCas12f1-v5.1 cleaved both DNA strands faster than WT AsCas12f1 (Figure 5D–F) (25).

To further identify the accelerated kinetic step (s), we revisited the smFRET assay with AsCas12f1-v5.1 (Figure 5G). Real-time FRET trajectories with this variant largely resembled those with WT AsCas12f1 and also showed the three FRET states of *S1*, *S2* and *S3*, as well as the subsequent loss of the acceptor signal in the optimal condition (Figures 3B and 5G). Statistical analysis revealed that there are no significant differences in the dwell times on the *S1* and *S3* states (*t_1_* and *t_3_*) between AsCas12f1-v5.1 and WT AsCas12f1, suggesting the kinetics of targeting DNA and processing out-of-protospacer DNA are comparable between the two nucleases (Figure 5H and J). However, the dwell time on the *S2* state (*t_2_*) was shortened with AsCas12f1-v5.1 (Figure 5I). In conclusion, AsCas12f1-v5.1 only accelerates the catalytic rate by improving the protospacer NTS DNA cleavage.

## Discussion

Employing ensemble and smFRET approaches, we explored the dynamic DNA association and dissociation of the miniature CRISPR-AsCas12f1 nuclease. Different from many other Cas effectors, AsCas12f1 engenders multiple DNA cleavage sites upon target recognition. Our work identified the intriguing bidirectional exonuclease activity involved in the sequential DNA cleavage of AsCas12f1, which ends up with the two cleavage sites on the NTS DNA (Figure 1). Consistently, a previous study demonstrated the out-of-protospacer DNA trimming when the AsCas12f1-sgRNA complex targeted a fully matched single-stranded DNA (19). The invisibility of the exonuclease activity of AsCas12f1 in the same study was probably because the ultrafast catalytic reaction under the optimal condition made the intermediate DNA cleavage products of AsCas12f1 undetectable (19). The bidirectional trimming of the NTS DNA has also been observed with SpCas9 (47). The difference between these two CRISPR-Cas proteins lies in the NTS DNA region cleaved: SpCas9 confined the exonucleolytic cleavage within the protospacer NTS DNA, and yet AsCas12f1 cleaved both the protospacer and out-of-protospacer NTS DNA (Figure 1). For this purpose, AsCas12f1 manages the unwinding and cleavage of the protospacer and out-of-protospacer DNA sequentially after the full-length crRNA–DNA hybridization (Figure 3). The capacity to unwind and/or edit out-of-protospacer DNA has been demonstrated with Type II-A Cas effectors, such as Nme2Cas9 and SpCas9 (48,49). Similarly, AsCas12f1 takes advantage of the out-of-protospacer DNA unwinding for both the exonucleolytic and endonucleolytic DNA cleavage beyond the protospacer (Figure 2A and B). Notably, the FRET signal in the *S3* state increased, indicating a reduced distance between the DNA region outside the protospacer and the RNP complex (Figure 3). Considering the only active RuvC domain in the dimerized AsCas2f1, it is plausible that AsCas12f1 gradually reels in the out-of-protospacer DNA and docks them in the domain. It is also possible that the shortened distance originates from the increased flexibility of the single-stranded TS DNA due to the NTS trimming beyond the protospacer.

**Figure 6.**
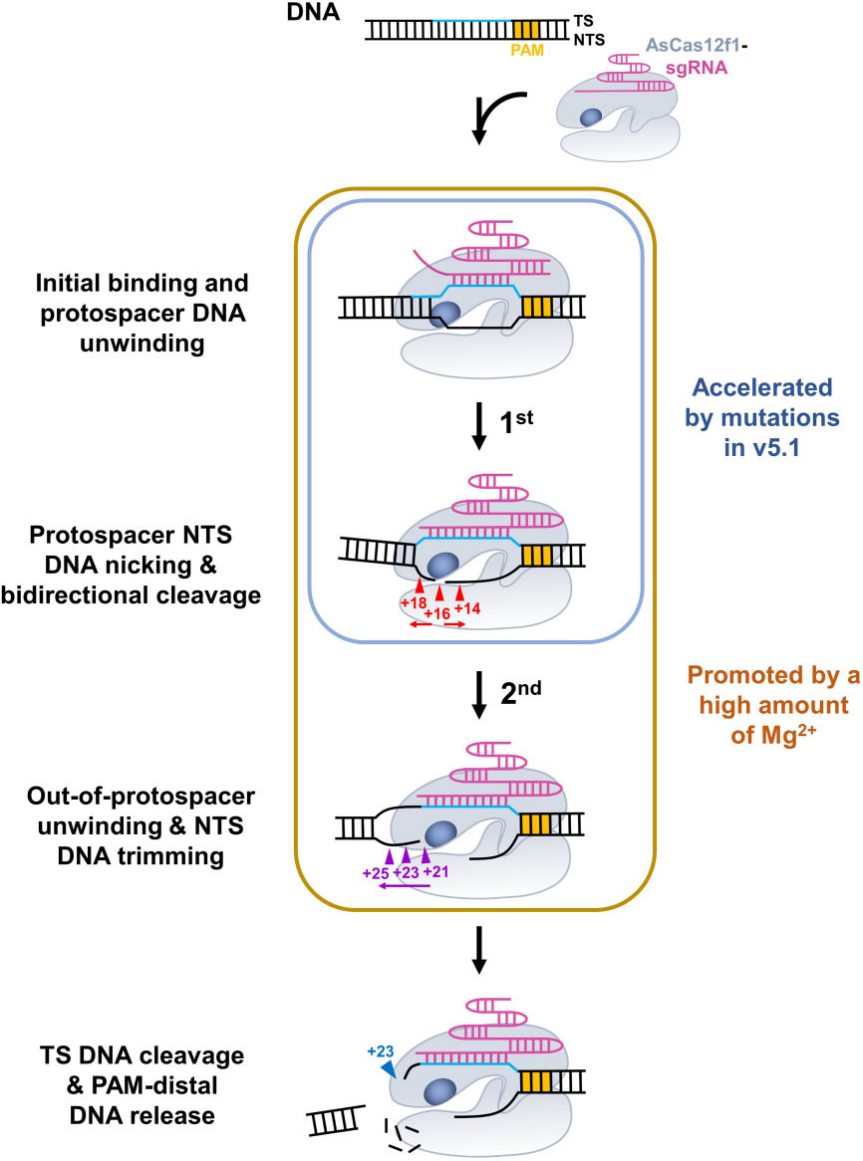
A mechanistic model of AsCas12f1 catalyzing the sequential DNA target cleavage. Upon PAM (yellow) recognition, AsCas12f1 first unwinds and cleaves the protospacer DNA (cyan) using the RuvC domain (dark blue). Then, DNA unwinding proceeds toward the out-of-protospacer region for AsCas12f1 to cleave beyond the protospacer. The exonuclease activity of AsCas12f1 is involved in the NTS DNA cleavage. The last endonucleolytic cleavage on the TS DNA releases the RNP complex from the PAM-distal DNA. The efficient protospacer and out-of-protospacer DNA unwinding and cleavage are ensured by a high amount of Mg^2+^ (brown rectangle). The mutations in the AsCas12f1-v5.1 variant accelerate the protospacer NTS DNA cleavage (blue rectangle). Colored triangles highlight the cleavage sites. The model is explained in detail in the text.

To summarize our findings, we propose a refined model for AsCas12f1-catalyzed DNA unwinding and cleavage (Figure 6): upon PAM recognition by the AsCas12f1-sgRNA complex, RNA–DNA hybridization proceeds from the PAM-proximal side to the PAM-distal side that promotes the protospacer DNA unwinding; the full-length heteroduplex formation allows for the initial NTS DNA nicking at the +16 position, followed by the bidirectional exonucleolytic cleavage to the +14 position and +18 position; after encountering the out-of-protospacer duplex DNA, the out-of-protospacer DNA unwinding takes place, which permits the digestion of the NTS DNA from the +21 position to the +25 position by AsCas12f1; finally, the nicking on the TS DNA at the +23 position immediately releases the RNP complex from the PAM-distal DNA (Figure 2C-F). The model may be generalizable to other CRISPR-Cas12 efforts that generate three cleavage sites on the DNA target, such as CnCas12f1 and SpaCas12f1 (16,17). Two dynamic pathways may account for the out-of-protospacer DNA unwinding: in one, AsCas12f1, powered by the chemical energy released from the hydrolysis of the nucleic acid chain, may reel in the NTS DNA and actively displace the TS DNA beyond the protospacer; alternatively, thermal fluctuation leads to the breathing of the DNA end generated by the NTS cleavage within the protospacer, and the transient DNA unwinding may be sufficient for AsCas12f1 to further the NTS DNA trimming. The second pathway is supported by the intrinsic instability of dsDNA flanking the 3′ end of gRNA found in the ternary Cas12a-gRNA–DNA complex (40,50). The exact pathway is worthy of further exploration.

In addition to the dynamic model, our work also provided a kinetic view of the catalytic reaction of AsCas12f1 and a molecular explanation of the enhanced insertion and deletion activity of AsCas12f1-v5.1. Based on our smFRET analysis, the cleavage reactions toward both DNA regions are promoted by Mg^2+^ (Figure 4). In the presence of a sufficient amount of Mg^2+^, AsCas12f1 quickly unwinds the protospacer DNA, and a stable R-loop structure is formed (*S1* to *S2* state). Under this condition, the overall DNA cleavage reaction of AsCas12f1 is dominantly limited by two steps regarding the interactions with the protospacer and out-of-protospacer DNA, respectively. On the other hand, under an insufficient amount of Mg^2+^, the partial crRNA–DNA hybridization restrains the protospacer NTS DNA trimming (Figure 4). Moreover, the tight coupling between out-of-protospacer DNA unwinding and cleavage also required Mg^2+^. The transient out-of-protospacer DNA unwinding can be reversed by DNA rewinding as the ssDNA is not digested promptly due to insufficient Mg^2+^ (Figure 4). These findings can be rationalized by the fact that the coordination of Mg^2+^ in the RuvC catalytic pocket of Cas12 is often required to create breaks in the dsDNA (51). The AsCas12f1-v5.1 variant is generated by mutating five amino acids (N70Q, K103R, A104R, S118A and D364R) to enhance the interaction between AsCas12f1 and DNA (25). According to the structural analysis, three amino acid mutations of the five interact with the protospacer TS and NTS DNA. Therefore, it is not surprising that AsCas12f1-v5.1 accelerates the kinetics of protospacer DNA cleavage (Figure 5). The N70Q mutation is supposed to strengthen the interaction between AsCas12f1 and the PAM, yet it seems not to aid in the DNA target association of AsCas12f1 (Figure 5H). In addition to RuvC, AsCas12f1 may require specific amino acids to unwind the out-of-protospacer DNA. Future attempts to enhance the editing efficiency of AsCas12f1 may focus on identifying and engineering vital amino acids specializing in the unwinding of the out-of-protospacer DNA.

## Supplementary Material

gkae989_Supplemental_File

## Data Availability

All data are available from the corresponding authors upon reasonable request and/or included in the manuscript as figure source data or supplementary data.

## References

[1] Wang J.Y. , Doudna J.A. CRISPR technology: a decade of genome editing is only the beginning. Science. 2023; 379:251–261. 10.1126/science.add8643 36656942

[2] Makarova K.S. , Wolf Y.I., Iranzo J., Shmakov S.A., Alkhnbashi O.S., Brouns S.J.J., Charpentier E., Cheng D., Haft D.H., Horvath P. et al. Evolutionary classification of CRISPR–Cas systems: a burst of class 2 and derived variants. Nat. Rev. Micro. 2019; 18:67–83. 10.1038/s41579-019-0299-x PMC8905525 31857715

[3] Wiedenheft B. , Sternberg S.H., Doudna J.A. RNA-guided genetic silencing systems in bacteria and archaea. Nature. 2012; 482:331–338. 22337052 10.1038/nature10886

[4] Stella S. , Alcón P., Montoya G. Class 2 CRISPR-Cas RNA-guided endonucleases: swiss Army knives of genome editing. Nat. Struct. Mol. Biol. 2017; 24:882–892. 29035385 10.1038/nsmb.3486

[5] Gasiunas G. , Barrangou R., Horvath P., Siksnys V. Cas9–crRNA ribonucleoprotein complex mediates specific DNA cleavage for adaptive immunity in bacteria. Proc. Natl Acad. Sci. U.S.A. 2012; 109:2579–2586. 22949671 10.1073/pnas.1208507109 PMC3465414

[6] Singh D. , Sternberg S.H., Fei J., Doudna J.A., Ha T. Real-time observation of DNA recognition and rejection by the RNA-guided endonuclease Cas9. Nat. Commun. 2016; 7:12778. 27624851 10.1038/ncomms12778 PMC5027287

[7] Sternberg S.H. , Redding S., Jinek M., Greene E.C., Doudna J.A. DNA interrogation by the CRISPR RNA-guided endonuclease Cas9. Nature. 2014; 507:62–67. 24476820 10.1038/nature13011 PMC4106473

[8] Singh D. , Mallon J., Poddar A., Wang Y., Tippana R., Yang O., Bailey S., Ha T. Real-time observation of DNA target interrogation and product release by the RNA-guided endonuclease CRISPR Cpf1 (Cas12a). Proc. Natl Acad. Sci. U.S.A. 2018; 115:5444–5449. 29735714 10.1073/pnas.1718686115 PMC6003496

[9] Anzalone A.V. , Koblan L.W., Liu D.R. Genome editing with CRISPR–Cas nucleases, base editors, transposases and prime editors. Nat. Biotechnol. 2020; 38:824–844. 32572269 10.1038/s41587-020-0561-9

[10] Liu G. , Lin Q., Jin S., Gao C. The CRISPR-Cas toolbox and gene editing technologies. Mol. Cell. 2022; 82:333–347. 34968414 10.1016/j.molcel.2021.12.002

[11] Chen K. , Wang Y., Zhang R., Zhang H., Gao C. CRISPR/Cas genome editing and precision plant breeding in agriculture. Annu. Rev. Plant Biol. 2019; 70:667–697. 30835493 10.1146/annurev-arplant-050718-100049

[12] Wang D. , Tai P.W.L., Gao G.P. Adeno-associated virus vector as a platform for gene therapy delivery. Nat. Rev. Drug Discov. 2019; 18:358–378. 30710128 10.1038/s41573-019-0012-9 PMC6927556

[13] Dong J.Y. , Fan P.D., Frizzell R.A. Quantitative analysis of the packaging capacity of recombinant adeno-associated virus. Hum. Gene Ther. 1996; 7:2101–2112. 8934224 10.1089/hum.1996.7.17-2101

[14] Harrington L.B. , Burstein D., Chen J.S., Paez-Espino D., Ma E., Witte I.P., Cofsky J.C., Kyrpides N.C., Banfield J.F., Doudna J.A. Programmed DNA destruction by miniature CRISPR-Cas14 enzymes. Science. 2018; 362:839–842. 30337455 10.1126/science.aav4294 PMC6659742

[15] Siksnys V. , Venclovas Č., Silanskas A., Gasior S., Djukanovic V., Paulraj S., Budre K., Zedaveinyte R., Hou Z., Young J.K. et al. PAM recognition by miniature CRISPR–Cas12f nucleases triggers programmable double-stranded DNA target cleavage. Nucleic. Acids. Res. 2020; 48:5016–5023. 32246713 10.1093/nar/gkaa208 PMC7229846

[16] Su M. , Li F., Wang Y., Gao Y., Lan W., Shao Z., Zhu C., Tang N., Gan J., Wu Z. et al. Molecular basis and engineering of miniature Cas12f with C-rich PAM specificity. Nat. Chem. Biol. 2023; 20:180–189. 37697004 10.1038/s41589-023-01420-4

[17] Wang Y. , Wang Y., Pan D., Yu H., Zhang Y., Chen W., Li F., Wu Z., Ji Q. Guide RNA engineering enables efficient CRISPR editing with a miniature Syntrophomonas palmitatica Cas12f1 nuclease. Cell Rep. 2022; 40:111418. 36170834 10.1016/j.celrep.2022.111418

[18] Wei Y. , Yang Z., Zong C., Wang B., Ge X., Tan X., Liu X., Tao Z., Wang P., Ma C. et al. trans single-stranded DNA cleavage via CRISPR/Cas14a1 activated by target RNA without destruction. Angew. Chem. Int. Ed. 2021; 60:24241–24247. 10.1002/anie.202110384 34553468

[19] Wu Z. , Zhang Y., Yu H., Pan D., Wang Y., Wang Y., Li F., Liu C., Nan H., Chen W. et al. Programmed genome editing by a miniature CRISPR-Cas12f nuclease. Nat. Chem. Biol. 2021; 17:1132–1138. 34475565 10.1038/s41589-021-00868-6

[20] Hino T. , Omura S.N., Nakagawa R., Togashi T., Takeda S.N., Hiramoto T., Tasaka S., Hirano H., Tokuyama T., Uosaki H. et al. An AsCas12f-based compact genome-editing tool derived by deep mutational scanning and structural analysis. Cell. 2023; 186:4920–4935. 37776859 10.1016/j.cell.2023.08.031

[21] Kim D.Y. , Lee J.M., Moon S.B., Chin H.J., Park S., Lim Y., Kim D., Koo T., Ko J.-H., Kim Y.-S. Efficient CRISPR editing with a hypercompact Cas12f1 and engineered guide RNAs delivered by adeno-associated virus. Nat. Biotechnol. 2021; 40:94–102. 34475560 10.1038/s41587-021-01009-z PMC8763643

[22] Kong X. , Zhang H., Li G., Wang Z., Kong X., Wang L., Xue M., Zhang W., Wang Y., Lin J. et al. Engineered CRISPR-OsCas12f1 and RhCas12f1 with robust activities and expanded target range for genome editing. Nat. Commun. 2023; 14:2046. 37041195 10.1038/s41467-023-37829-7 PMC10090079

[23] Xin C. , Yin J., Yuan S., Ou L., Liu M., Zhang W., Hu J. Comprehensive assessment of miniature CRISPR-Cas12f nucleases for gene disruption. Nat. Commun. 2022; 13:5623. 36153319 10.1038/s41467-022-33346-1 PMC9509373

[24] Wu T. , Liu C., Zou S., Lyu R., Yang B., Yan H., Zhao M., Tang W. An engineered hypercompact CRISPR-Cas12f system with boosted gene-editing activity. Nat. Chem. Biol. 2023; 19:1384–1393. 37400536 10.1038/s41589-023-01380-9 PMC10625714

[25] Wu Z. , Liu D., Pan D., Yu H., Shi J., Ma J., Fu W., Wang Z., Zheng Z., Qu Y. et al. Structure and engineering of miniature *Acidibacillus sulfuroxidans* Cas12f1. Nat. Catal. 2023; 6:695–709.

[26] Sun B. , Wang M.D. Single-molecule optical-trapping techniques to study molecular mechanisms of a replisome. Methods Enzymol. 2017; 582:55–84. 28062045 10.1016/bs.mie.2016.08.001

[27] Ye S.S. , Chen Z.T., Zhang X., Li F.F., Guo L.J., Hou X.M., Wu W.Q., Wang J., Liu C., Zheng K. et al. Proximal single-stranded RNA destabilizes Human telomerase RNA G-quadruplex and induces its distinct conformers. J. Phys. Chem. Lett. 2021; 12:3361–3366. 33783224 10.1021/acs.jpclett.1c00250

[28] Jia X. , Li Y., Wang T., Bi L., Guo L., Chen Z., Zhang X., Ye S., Chen J., Yang B. et al. Discrete RNA–DNA hybrid cleavage by the EXD2 exonuclease pinpoints two rate-limiting steps. EMBO J. 2022; 42:e111703. 36326837 10.15252/embj.2022111703 PMC9811613

[29] Roy R. , Hohng S., Ha T. A practical guide to single-molecule FRET. Nat. Methods. 2008; 5:507–516. 18511918 10.1038/nmeth.1208 PMC3769523

[30] Zhang Q. , Chen Z.T., Sun B. Molecular mechanisms of *Streptococcus pyogene**s* Cas9: a single-molecule perspective. Biophys. Rep. 2021; 7:475–489. 37288365 10.52601/bpr.2021.210021 PMC10210056

[31] Juette M.F. , Terry D.S., Wasserman M.R., Altman R.B., Zhou Z., Zhao H., Blanchard S.C. Single-molecule imaging of non-equilibrium molecular ensembles on the millisecond timescale. Nat. Methods. 2016; 13:341–344. 26878382 10.1038/nmeth.3769 PMC4814340

[32] McKinney S.A. , Joo C., Ha T. Analysis of single-molecule FRET trajectories using hidden Markov modeling. Biophys. J. 2006; 91:1941–1951. 16766620 10.1529/biophysj.106.082487 PMC1544307

[33] Yardimci H. , Loveland A.B., van Oijen A.M., Walter J.C. Single-molecule analysis of DNA replication in Xenopus egg extracts. Methods. 2012; 57:179–186. 22503776 10.1016/j.ymeth.2012.03.033 PMC3427465

[34] Hall M.A. , Shundrovsky A., Bai L., Fulbright R.M., Lis J.T., Wang M.D. High-resolution dynamic mapping of histone-DNA interactions in a nucleosome. Nat. Struct. Mol. Biol. 2009; 16:124–129. 19136959 10.1038/nsmb.1526 PMC2635915

[35] Smith S.B. , Cui Y., Bustamante C. Overstretching B-DNA: the elastic response of individual double-stranded and single-stranded DNA molecules. Nature. 1996; 271:795–799. 10.1126/science.271.5250.795 8628994

[36] Pacesa M. , Loeff L., Querques I., Muckenfuss L.M., Sawicka M., Jinek M. R-loop formation and conformational activation mechanisms of Cas9. Nature. 2022; 609:191–196. 36002571 10.1038/s41586-022-05114-0 PMC9433323

[37] Jiang F.G. , Taylor D.W., Chen J.S., Kornfeld J.E., Zhou K.H., Thompson A.J., Nogales E., Doudna J.A. Structures of a CRISPR-Cas9 R-loop complex primed for DNA cleavage. Science. 2016; 351:867–871. 26841432 10.1126/science.aad8282 PMC5111852

[38] Stella S. , Alcón P., Montoya G. Structure of the Cpf1 endonuclease R-loop complex after target DNA cleavage. Nature. 2017; 546:559–563. 28562584 10.1038/nature22398

[39] Schmidt K.S. Application of locked nucleic acids to improve aptamer in vivo stability and targeting function. Nucleic. Acids. Res. 2004; 32:5757–5765. 15509871 10.1093/nar/gkh862 PMC528785

[40] Son H. , Park J., Hwang I., Jung Y., Bae S., Lee S. Mg2+-dependent conformational rearrangements of CRISPR-Cas12a R-loop complex are mandatory for complete double-stranded DNA cleavage. Proc. Natl Acad. Sci. U.S.A. 2021; 118:e2113747118. 34853172 10.1073/pnas.2113747118 PMC8670479

[41] Braasch D.A. , Corey D.R. Locked nucleic acid (LNA): fine-tuning the recognition of DNA and RNA. Chem. Biol. 2001; 8:1–7. 11182314 10.1016/s1074-5521(00)00058-2

[42] Vester B. , Wengel J. LNA (Locked nucleic acid): high-affinity targeting of complementary RNA and DNA. Biochemistry. 2004; 43:13233–13241. 15491130 10.1021/bi0485732

[43] Heller I. , Hoekstra T.P., King G.A., Peterman E.J.G., Wuite G.J.L. Optical tweezers analysis of DNA–Protein complexes. Chem. Rev. 2014; 114:3087–3119. 24443844 10.1021/cr4003006

[44] Zhang Q. , Chen Z., Wang F., Zhang S., Chen H., Gu X., Wen F., Jin J., Zhang X., Huang X. et al. Efficient DNA interrogation of SpCas9 governed by its electrostatic interaction with DNA beyond the PAM and protospacer. Nucleic. Acids. Res. 2021; 49:12433–12444. 34850124 10.1093/nar/gkab1139 PMC8643646

[45] Zhang Q , ZhangS W.F., Jin J, Bi L, Lu Y, Li M, Xi XG, Huang X, Shen B, Sun B The post-PAM interaction of RNA-guided spCas9 with DNA dictates its target binding and dissociation. Sci. Adv. 2019; 5:e9807. 10.1126/sciadv.aaw9807 PMC6853773 31763447

[46] Zhang S. , Zhang Q., Hou X.M., Guo L., Wang F., Bi L., Zhang X., Li H.H., Wen F., Xi X.G. et al. Dynamics of *S**taphylococcus aure**us* Cas9 in DNA target association and dissociation. EMBO Rep. 2020; 21:e50184. 32790142 10.15252/embr.202050184 PMC7534634

[47] Stephenson A.A. , Raper A.T., Suo Z.C. Bidirectional degradation of DNA cleavage products catalyzed by CRISPR/Cas9. J. Am. Chem. Soc. 2018; 140:3743–3750. 29461055 10.1021/jacs.7b13050

[48] Chen Z. , Li X., Zhang Q., Sun W., Song X., Zhang X., Huang X., Sun B. Enlarged DNA unwinding by Nme2Cas9 permits a broadened base editing window beyond the protospacer. Sci. China Life Sci. 2024; 67:424–427. 37606848 10.1007/s11427-023-2384-9

[49] Thuronyi B.W. , Koblan L.W., Levy J.M., Yeh W.-H., Zheng C., Newby G.A., Wilson C., Bhaumik M., Shubina-Oleinik O., Holt J.R. et al. Continuous evolution of base editors with expanded target compatibility and improved activity. Nat. Biotechnol. 2019; 37:1070–1079. 31332326 10.1038/s41587-019-0193-0 PMC6728210

[50] Cofsky J.C. , Karandur D., Huang C.J., Witte I.P., Kuriyan J., Doudna J.A. CRISPR-Cas12a exploits R-loop asymmetry to form double-strand breaks. eLife. 2020; 9:e55143. 32519675 10.7554/eLife.55143 PMC7286691

[51] Huang X. , Sun W., Cheng Z., Chen M., Li X., Wang J., Sheng G., Gong W., Wang Y. Structural basis for two metal-ion catalysis of DNA cleavage by Cas12i2. Nat. Commun. 2020; 11:5241. 33067443 10.1038/s41467-020-19072-6 PMC7567891

