# Supplementary material for: CRISPR-AsCas12f1 couples out-of-protospacer DNA unwinding with exonuclease activity in the sequential target cleavage
Source: Nucleic Acids Res. 2024 Nov 12;52(22):14030–42. doi: 10.1093/nar/gkae989 (PMC11662939; doi:10.1093/nar/gkae989)
Supplement: gkae989_Supplemental_File [file gkae989_supplemental_file.pdf]

Supplementary Materials

CRISPR-AsCas12f1 couples out-of-protospacer DNA unwinding with exonuclease activity in the sequential target cleavage

Xiaoxuan Song[1,†], Ziting Chen[1,2,†], Wenjun Sun[1], Hao Yang[3], Lijuan Guo[1], Yilin Zhao[1], Yanan Li[1], Zhiyun Ren[1,4,5], Jin Shi[6], Cong Liu[5,7], Peixiang Ma[8], Xingxu Huang[9], Quanjiang Ji[6,10], and Bo Sun[1,10,*]

[1]School of Life Science and Technology, ShanghaiTech University, Shanghai 201210, China, [2] ENT Institute and Department of Otorhinolaryngology, Eye & ENT Hospital, State Key Laboratory of Medical Neurobiology and MOE Frontiers Center for Brain Science, NHC Key Laboratory of Hearing Medicine, Institutes of Biomedical Sciences, Fudan University, Shanghai 200031, China, [3]School of Life Sciences and Biotechnology, Shanghai Jiao Tong University, Shanghai, 200240, China, [4]CAS Center for Excellence in Molecular Cell Science, Shanghai Institute of Biochemistry and Cell Biology, Chinese Academy of Sciences, Shanghai 200031, China, [5]University of Chinese Academy of Sciences, Beijing 100049, China, [6]School of Physical Science and Technology, ShanghaiTech University, Shanghai 201210, China, [7]Interdisciplinary Research Center on Biology and Chemistry, Shanghai Institute of Organic Chemistry, Chinese Academy of Sciences, Shanghai 201210, China, [8]Shanghai Key Laboratory of Orthopedic Implants, Department of Orthopedic Surgery, Shanghai Ninth People's Hospital, Shanghai Jiao Tong University School of Medicine, Shanghai, 200025, China, [9]Zhejiang lab, Hangzhou, Zhejiang 311121, China, [10]Gene Editing Center, ShanghaiTech University, Shanghai 201210, China.

*To whom correspondence should be addressed. Tel:+86 21 2068 4536; Fax: +86 21 2068 5430; Email: sunbo@shanghaitech.edu.cn

[†]The first two authors should be regarded as Joint First Authors.

This file includes

Figures S1 – S15
Table S1
Reference S1

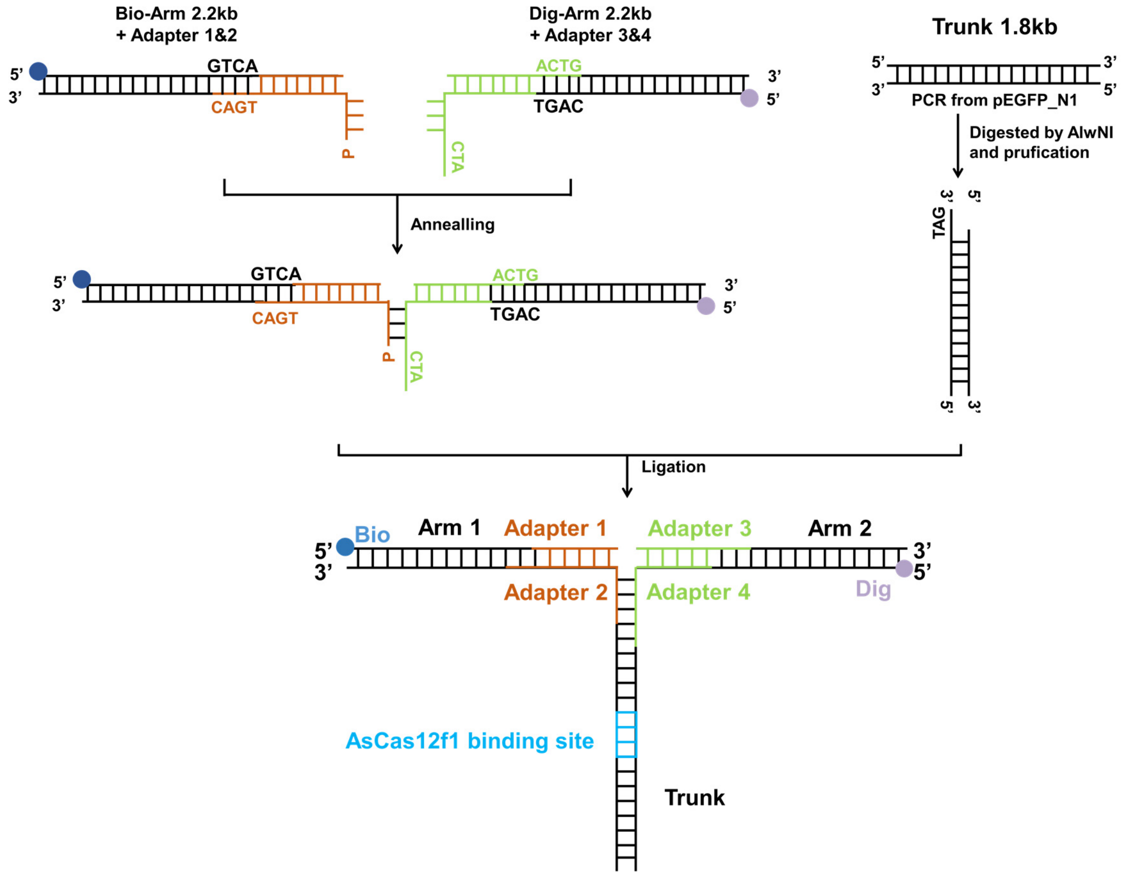

Figure S1. DNA template design for single-molecule DNA unzipping assay. The DNA construct consists of three DNA segments: two arms and a trunk, linked through four short adapters (Table S1) (1). The trunk DNA contains a single AsCas12f1-sgRNA binding site (blue). Two arms of the DNA construct were attached to a trapped microsphere and a microscope coverslip, respectively. The unzipping of the trunk DNA is initiated from the fork due to a gap between adapters 1 and 3.

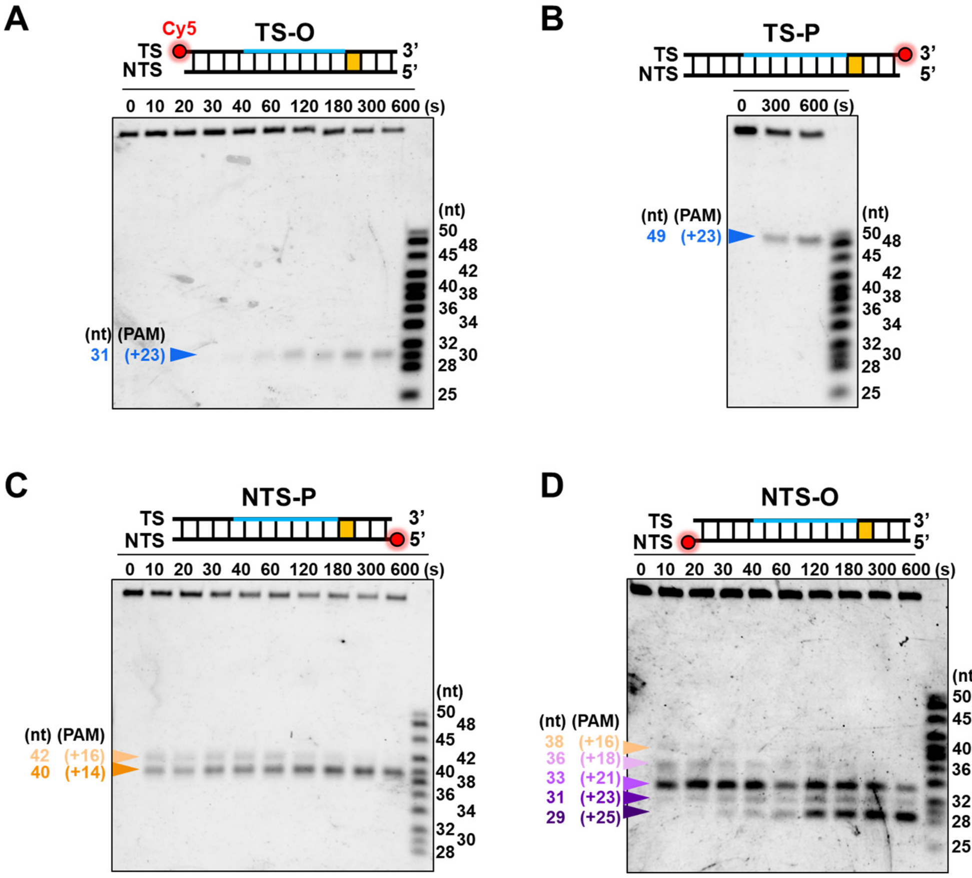

Figure S2. Determination of DNA cleavage sites of AsCas12f1. A. A representative gel of AsCas12f1-catalyzed DNA cleavage toward the TS-O substrate. **B.** A representative gel of AsCas12f1-catalyzed DNA cleavage toward the TS-P substrate. **C.** A representative gel of AsCas12f1-catalyzed DNA cleavage toward the NTS-P substrate. **D.** A representative gel of AsCas12f1-catalyzed DNA cleavage toward the NTS-O substrate. Markers spaced by 2 nt were used to determine the precise cleavage sites.

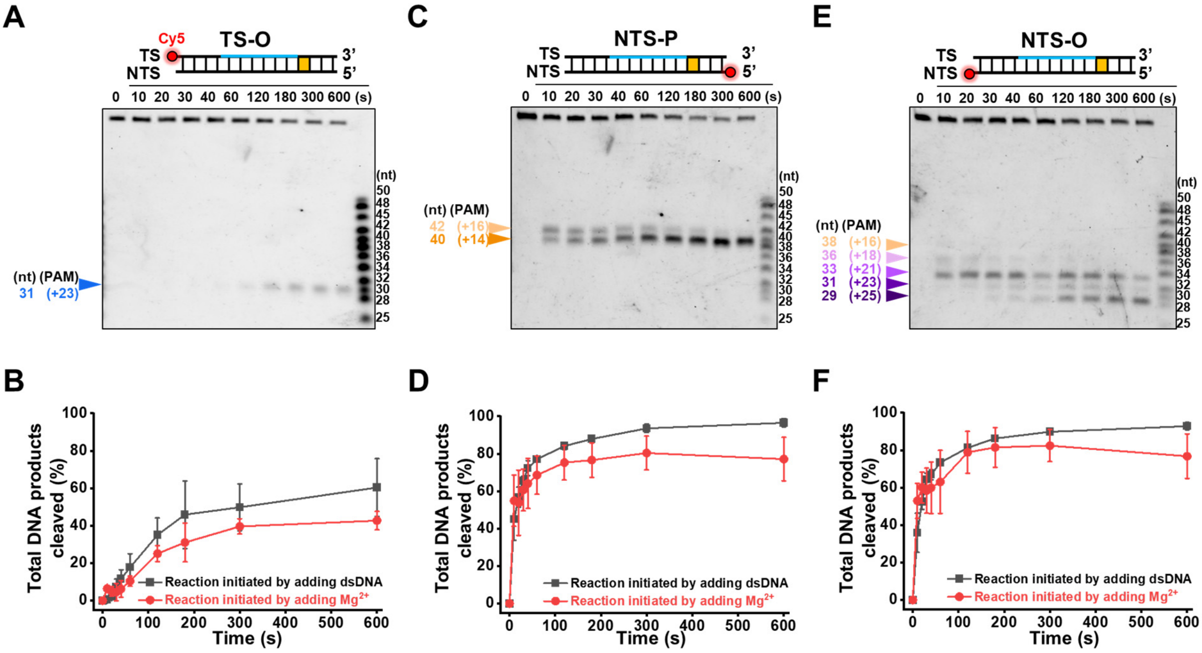

Figure S3: AsCas12f1-mediated DNA cleavage upon the addition of Mg^{2+}. A. C. E. Representative gels of AsCas12f1-catalyzed DNA cleavage toward the TS-O, NTS-P, and NTS-O substrates, respectively. The AsCas12f1-gRNA complex was pre-mixed with the dsDNA substrates. The addition of Mg^{2+} initiates the reaction. **B. D. F.** Quantification of the corresponding cleavage products as a function of time. Compared with the cleavage reactions triggered by lastly adding dsDNA (black), the cleavage rates are slightly slower when initiated by adding Mg^{2+} (red).

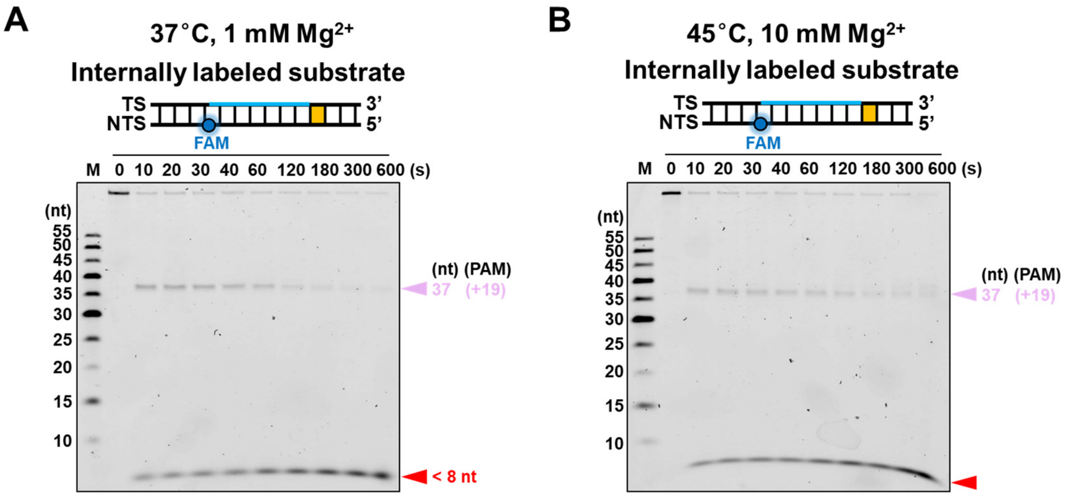

Figure S4. AsCas12f1-mediated DNA cleavage toward the internally labeled DNA substrate. A. A representative gel of AsCas12f1-catalyzed DNA cleavage toward the internally labeled substrate at 37°C and 1 mM Mg^{2+}. **B.** A representative gel of AsCas12f1-catalyzed DNA cleavage toward the internally labeled substrate at 45°C and 10 mM Mg^{2+}. The fluorescence dye (FAM) was labeled at the +20 position on the NTS DNA within the two NTS DNA cleavage sites (Table S1).

The DNA products of less than 8 nt support the exonuclease activity of AsCas12f1 on the NTS DNA under both experimental conditions.

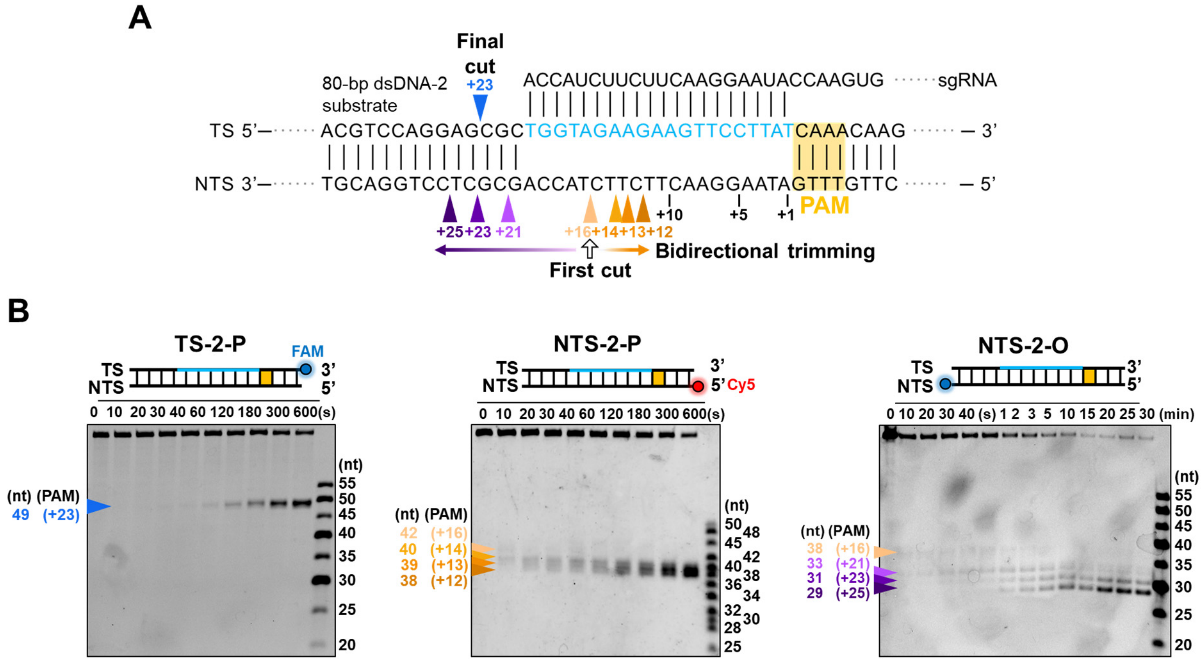

Figure S5. AsCas12f1-catalyzed target DNA-2 cleavage with both endonuclease and exonuclease activities. A. A map for the cleavage pattern of AsCas12f1 toward the dsDNA-2 substrate (Table S1). The protospacer DNA and the PAM are colored cyan and yellow, respectively. Triangles indicate the cleavage sites and directionality. **B.** Representative gels of AsCas12f1-catalyzed cleavage toward the TS-2-P, NTS-2-P, and NTS-2-O substrates (Table S1).

The time-sensitive, ladder-like cleavage pattern seen with the DNA-2 substrates suggests the inherent bidirectional NTS exonucleolytic cleavage of AsCas12f1, even though the cleavage sites varied slightly.

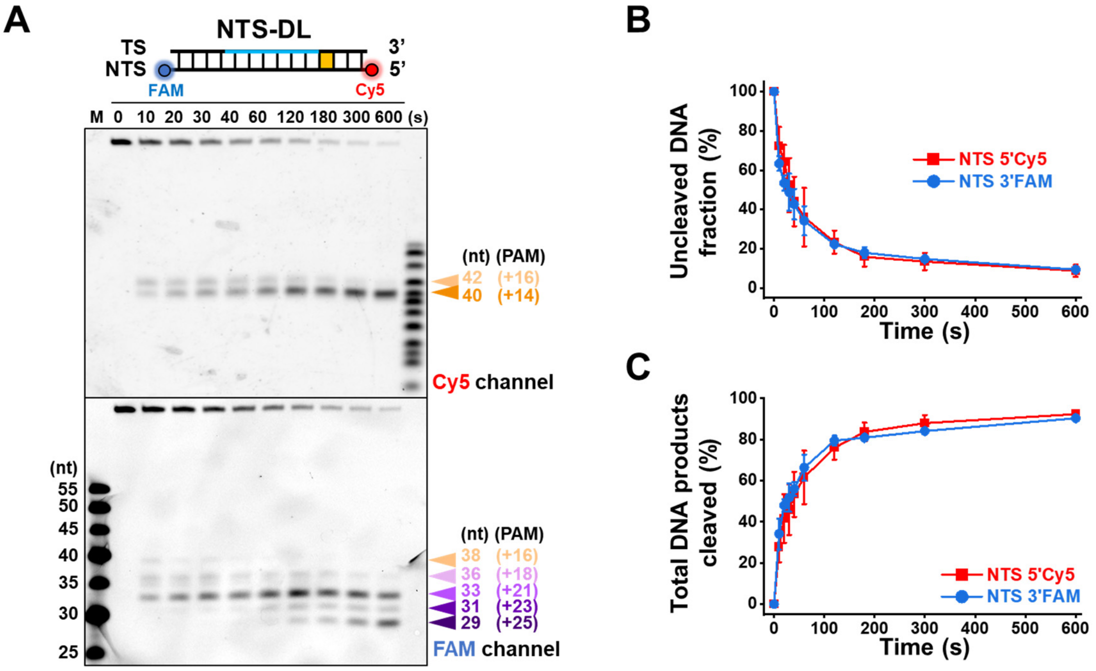

Figure S6. AsCas12f1 cleaves target DNA with dual-end fluorescence labeling. A. Representative gels of AsCas12f1-catalyzed cleavage on the NTS-DL DNA substrates. The 5' and 3' ends of the NTS of this substrate are labeled with Cy5 and FAM, respectively. **B.** Statistical analysis of the uncleaved DNA substrates in the two gels. Error bars represent the S.D. of three replicates. **C.** Statistical analysis of the cleaved DNA substrates in the two gels. Error bars represent the S.D. of three replicates.

The cleaved and uncleaved DNA followed similar trends in the two gels, suggesting that a single AsCas12f1 protein executed the bidirectional exonucleolytic cleavage.

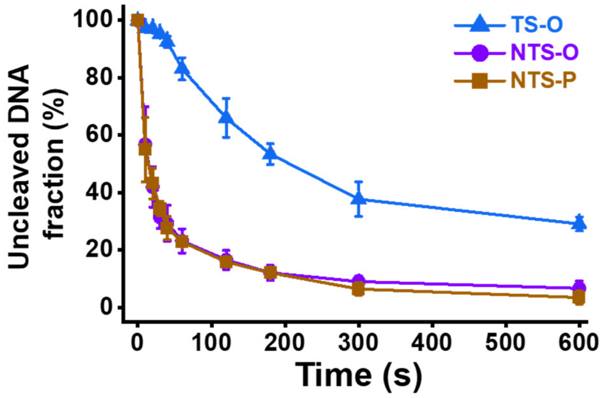

Figure S7. Fractions of DNA substrates not cleaved by AsCas12f1 as a function of time. Statistical analysis of the TS-O, NTS-O, and NTS-P DNA substrates not cleaved by AsCas12f1 from Figure 1B, 1D, & 1F. Error bars represent the S.D. of three replicates.

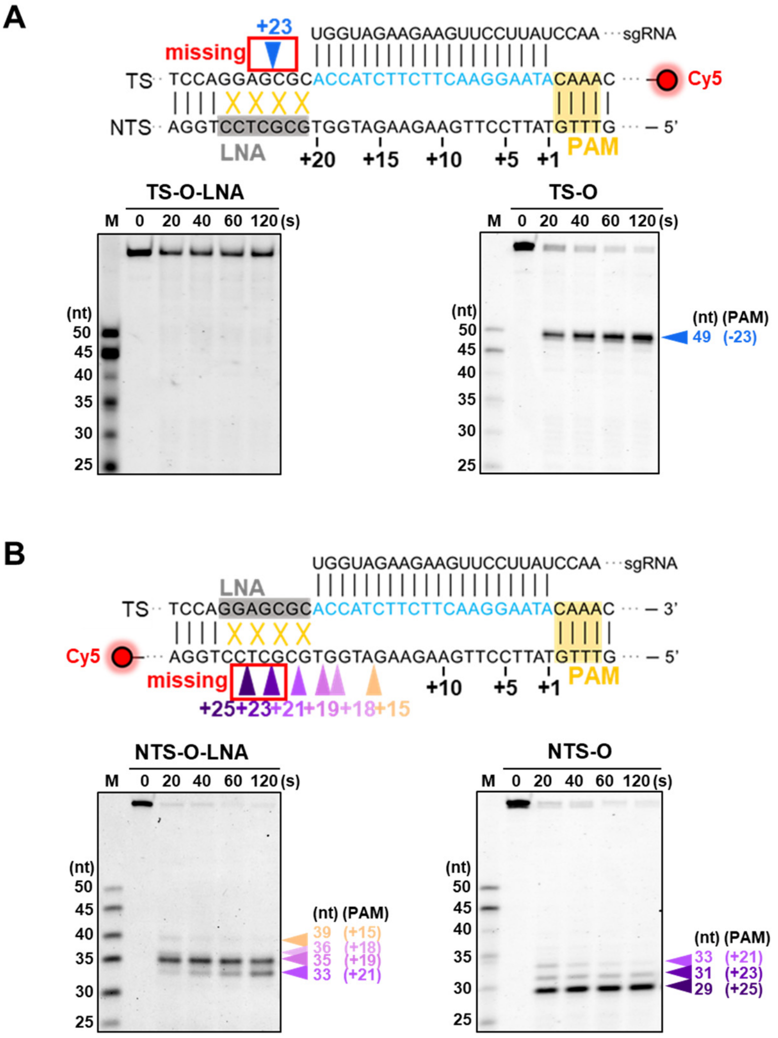

Figure S8. Cleavage of LNA-modified DNA substrates by AsCas12f1 under the optimal condition. A. The sequence pattern diagrams are shown above the representative gels of AsCas12f1-catalyzed DNA cleavage toward the TS-O-LNA and TS-O substrates. **B.** The sequence pattern diagrams are shown above the representative gels of AsCas12f1-catalyzed DNA cleavage toward the NTS-O-LNA and NTS-O substrates. The protospacer DNA and the PAM are colored cyan and yellow, respectively. Colored triangles indicate the cleavage sites, and those framed indicate the missing ones.

These DNA cleavage experiments were carried out under 10 mM Mg^{2+} at 45°C. Under this condition, three cleavage sites beyond the protospacer are still missing on the LNA-modified substrates.

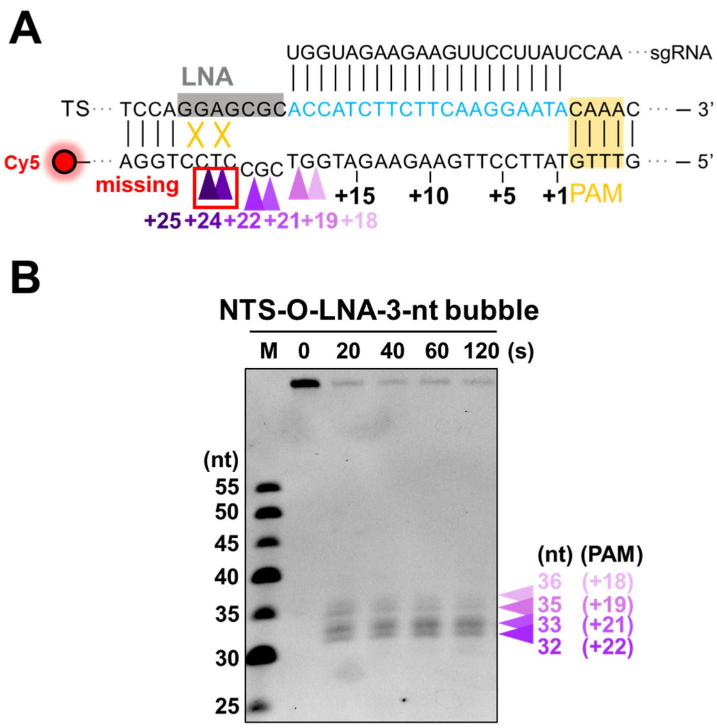

Figure S9. Cleavage of the LNA-modified bubbled DNA substrates by AsCas12f1. A. A map for the LNA-modified bubble DNA cleavage pattern of AsCas12f1. The protospacer DNA and the PAM are colored cyan and yellow, respectively. Colored triangles indicate the cleavage sites, and those framed indicate the missing ones. Three base pairs close to the protospacer (+21 - +23) are unpaired. **B.** A representative gel of AsCas12f1-catalyzed DNA cleavage toward the NTS-O-LNA-3-nt bubble substrate. Colored triangles indicate the cleavage sites and directionality.

The unpaired bases from +21 to +23 allow AsCas12f1 to cleave within this region, and yet the paired LNA bases prevent AsCas12f1 from cleaving at the +24 - +27 positions. Therefore, the cleavage sites are correlated with the DNA unwinding status, and pre-separation of the out-of-protospacer is a prerequisite for AsCas12f1-meidated DNA trimming.

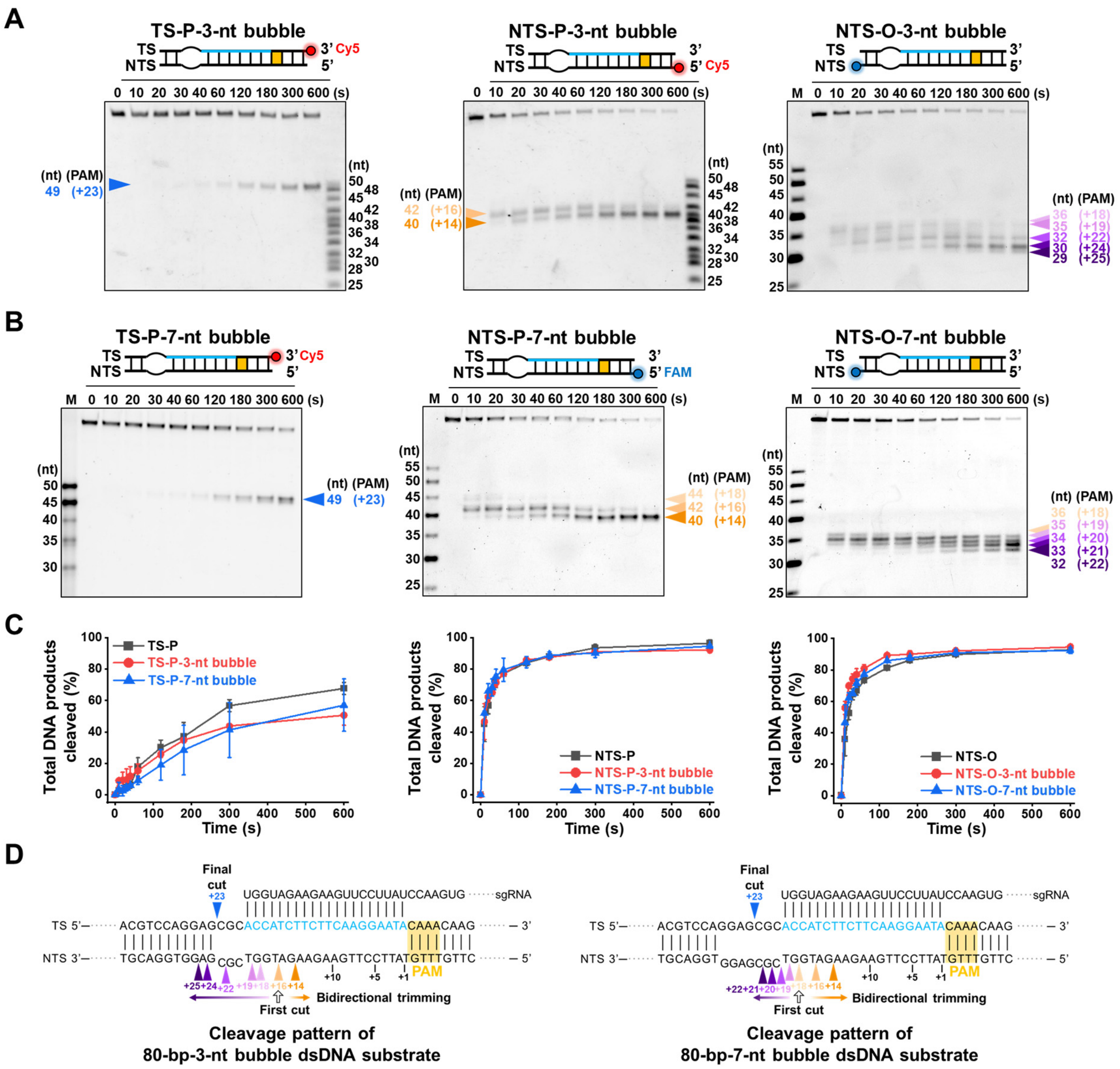

Figure S10. AsCas12f1-mediated DNA cleavage toward the 3-nt and 7-nt bubble DNA substrates. A. Representative gels of AsCas12f1-catalyzed DNA cleavage toward the TS-P-3-nt bubble, NTS-P-3-nt bubble, and NTS-O-3-nt bubble substrates. The nucleotides at +21 - +23 are mismatched. **B.** Representative gels of AsCas12f1-catalyzed DNA cleavage toward the TS-P-7-nt bubble, NTS-P-7-nt bubble, and NTS-O-7-nt bubble substrates. The nucleotides at +21 - +27 are mismatched. **C.** Quantitation of the cleavage products of bubbled DNA substrates by AsCas12f1. Error bars represent the S.D. of three replicates. **D.** Maps for the bubble DNA cleavage pattern of AsCas12f1. The protospacer DNA and the PAM are colored cyan and yellow, respectively. Colored triangles indicate the cleavage sites and directionality.

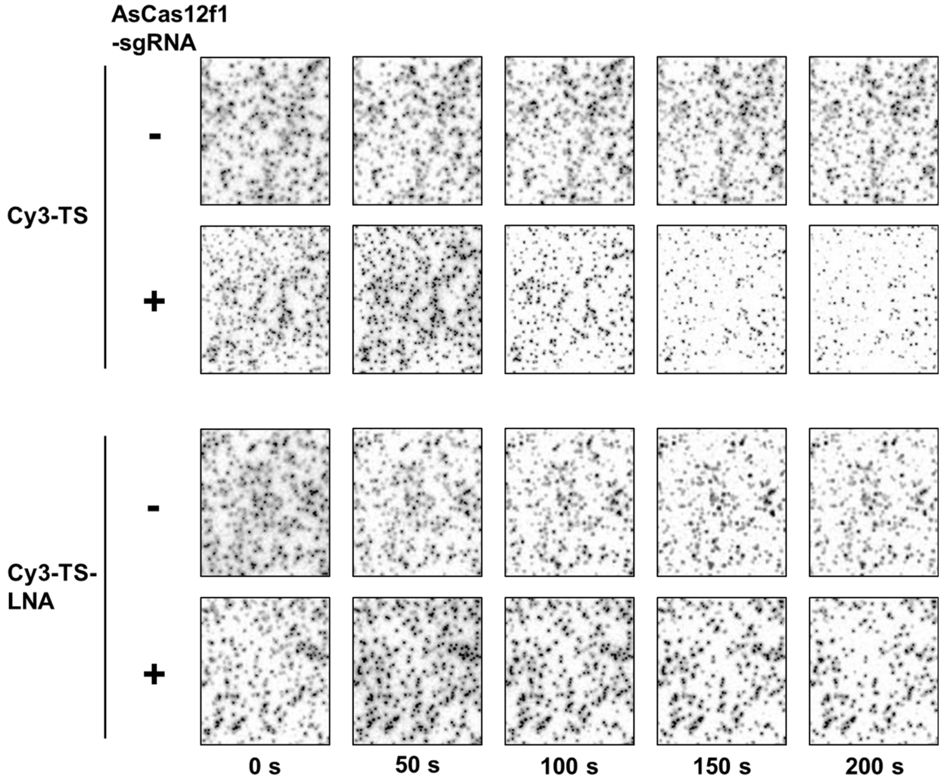

Figure S11. The fluorescence signals on Cy3-labeled substrates at the indicated time. Representative images display the fluorescence signal of the Cy3-TS or Cy3-TS-LNA substrates in the absence and presence of AsCas12f1-sgRNA at the indicated times.

The gradually disappearing fluorescence signal suggests that AsCas12f1-sgRNA cleaved the Cy3-TS and released the PAM-distal DNA, a catalytic reaction prohibited on the Cy3-TS-LNA substrate.

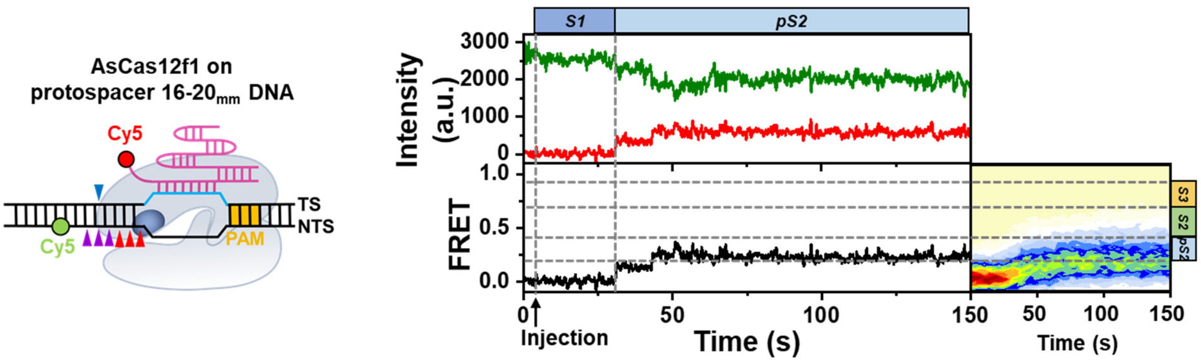

Figure S12. A representative smFRET trajectory of AsCas12f1-gRNA binding onto a partially matched dsDNA template. A cartoon illustrates the smFRET experiment to detect DNA binding and cleavage by AsCas12f1 complexed with a partially matched sgRNA from 16 to 20 positions (16-20$_{mm}$) (Table S1). Colored triangles indicate the cleavage sites at different locations. A representative FRET trajectory is shown on the right. FRET contour plots are shown on the right of the real-time trajectories.

Upon AsCas12f1-sgRNA associated with the partially matched DNA target, a FRET value of ~ 0.2 appeared, which is lower than that with a fully matched DNA target. This low FRET status is likely due to the partial hybridization between crRNA and DNA. Therefore, the FRET value of 0.5 detected with the fully matched DNA target is likely a result of the full-length hybridization between crRNA and DNA.

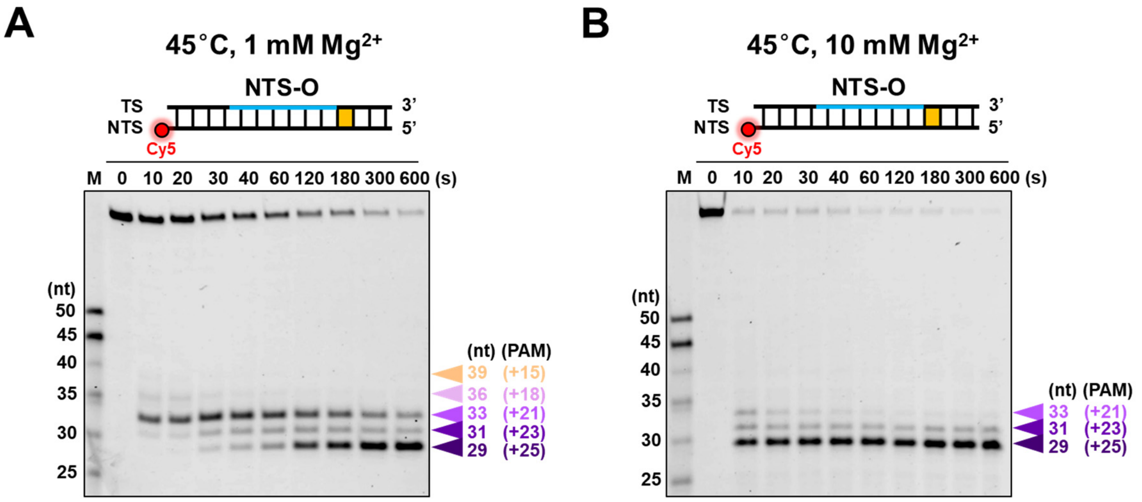

Figure S13. Cleavage of the NTS by AsCas12f1 under varied Mg^{2+} concentrations. A. A representative gel of AsCas12f1-catalyzed DNA cleavage toward the NTS-O substrate at 45°C and 1 mM Mg^{2+}. **B.** A representative gel of AsCas12f1-catalyzed DNA cleavage toward the NTS-O substrate at 45°C and 10 mM Mg^{2+}. Colored triangles indicate the cleavage sites.

Based on these gels, a high amount of Mg^{2+} promotes the AsCas12f1-catalyzed NTS DNA cleavage. The ladder-like cleavage pattern is also detectable under the optimal cleavage condition.

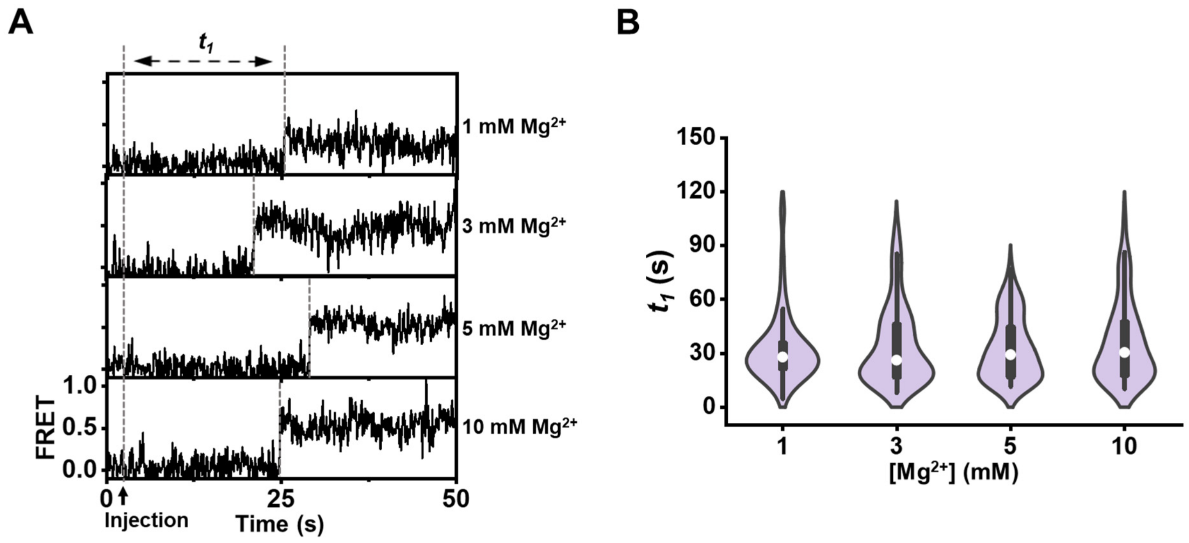

Figure S14. Representative FRET trajectories and statistical comparisons of the dwell times on the *S1* state under different Mg^{2+} concentrations. A. Representative FRET trajectories of AsCas12f1 targeting DNA under varied Mg^{2+} concentration. **B.** Statistics of the dwell time (*t1*) on the *S1* states under varied Mg^{2+} concentration. In the violin plots, white dots represent the mean, and black bars show the interquartile range (IQR) (thick bars) and 1.5 times IQR (thin bars) (n = 74, 76, 66, and 63 from left to right).

The comparable dwell times on the *S1* state suggest that the effect of Mg^{2+} on the DNA target binding of AsCas12f1 is negligible.

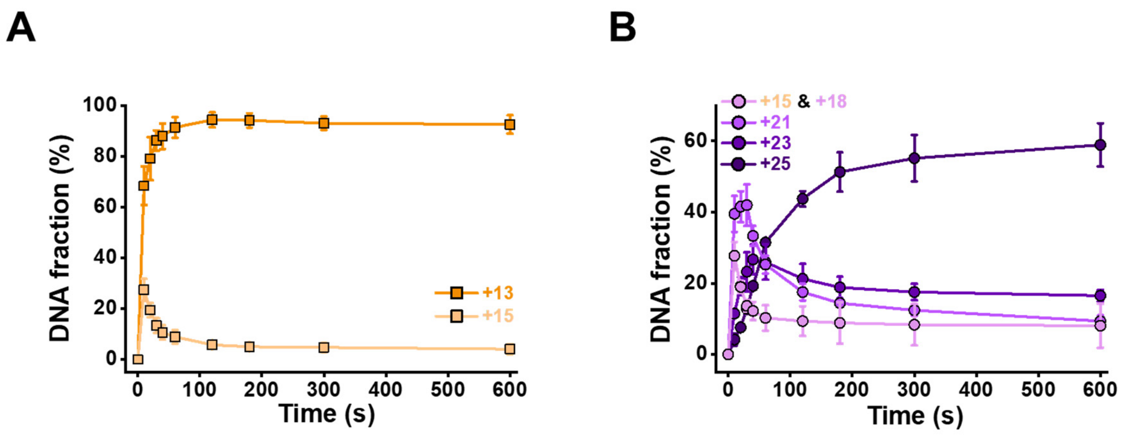

Figure S15. Quantifications of the NTS DNA products cleaved by AsCas12f1-v5.1. A. Quantifications of DNA cleavage products (+13 and +15) from Figure 5B as a function of time. **B.** Quantifications of DNA cleavage products (+15 plus +18, +21, +23, and +25) from Figure 5B as a function of time. Error bars represent the S.D. of three replicates.

Table S1. Sequences of DNA and RNA used.

Figures	Name	Sequence
Figures 1, 5, S2, S3, S6, S13	80-bp dsDNA	5’-ACGCACCAGAAACGAAGGCTACGTCCAGGAGCGCACCATCTTCTTCAAGGAATACAAACAAGCTAAGCACAACGTCGTCT-3’
		5’-AGACGACGTTGTGCTTAGCTTGTTTGTATTCCTTGAAGAAGATGGTGCGCTCCTGGACGTAGCCTTCGTTTCTGGTGCGT-3’
Figures 2 & S8	TS-O-LNA	5’-ACGCACCAGAAACGAAGGCTACGTCCAGGAGCGCACCATCTTCTTCAAGGAATACAAACAAGCTAAGCACAACGTCGTCT-3’
		5’-AGACGACGTTGTGCTTAGCTTGTTTGTATTCCTTGAAGAAGATGGTGCGCTCCTGGACGTAGCCTTCGTTTCTGGTGCGT-3’
	NTS-O-LNA	5’-ACGCACCAGAAACGAAGGCTACGTCCAGGAGCGCACCATCTTCTTCAAGGAATACAAACAAGCTAAGCACAACGTCGTCT-3’
		5’-AGACGACGTTGTGCTTAGCTTGTTTGTATTCCTTGAAGAAGATGGTGCGCTCCTGGACGTAGCCTTCGTTTCTGGTGCGT-3’
Figure S9	NTS-O-LNA-3 nt bubble	5’-ACGCACCAGAAACGAAGGCTACGTCCAGGAGCGCACCATCTTCTTCAAGGAATACAAACAAGCTAAGCACAACGTCGTCT-3’
		5’-AGACGACGTTGTGCTTAGCTTGTTTGTATTCCTTGAAGAAGATGGTCGCCTCCTGGACGTAGCCTTCGTTTCTGGTGCGT-3’
Figure S10	3 nt bubble DNA	5’-ACGCACCAGAAACGAAGGCTACGTCCAGGAGCGCACCATCTTCTTCAAGGAATACAAACAAGCTAAGCACAACGTCGTCT-3’
		5’-AGACGACGTTGTGCTTAGCTTGTTTGTATTCCTTGAAGAAGATGGTCGCCTCCTGGACGTAGCCTTCGTTTCTGGTGCGT-3’
	7 nt bubble DNA	5’-ACGCACCAGAAACGAAGGCTACGTCCAGGAGCGCACCATCTTCTTCAAGGAATACAAACAAGCTAAGCACAACGTCGTCT-3’
		5’-AGACGACGTTGTGCTTAGCTTGTTTGTATTCCTTGAAGAAGATGGTCGCGAGGTGGACGTAGCCTTCGTTTCTGGTGCGT-3’
Figure 2 & S11	Cy3-TS	5’-biotin-ACGCACCAGAAACGAAGGCTACGTCCAGGAGCGCACCA/iCy3dT/CTTCTTCAAGGAATACAAACAAGCTAAGCACAACGTCGTCT-3’
		5’-AGACGACGTTGTGCTTAGCTTGTTTGTATTCCTTGAAGAAGATGGTGCGCTCCTGGACGTAGCCTTCGTTTCTGGTGCGT-3’
	Cy3-TS-LNA	5’-biotin-ACGCACCAGAAACGAAGGCTACGTCCAGGAGCGCACCA/iCy3dT/CTTCTTCAAGGAATACAAACAAGCTAAGCACAACGTCGTCT-3’
		5’-AGACGACGTTGTGCTTAGCTTGTTTGTATTCCTTGAAGAAGATGGTGCGCTCCTGGACGTAGCCTTCGTTTCTGGTGCGT-3’
Figures 3, 4, 5	Unmodified DNA	5’-ACGCACCAGAAACGAAGGCTACGTCCAGGAGCGCACCATCTTCTTCAAGGAATACAAACAAGCTAAGCACAACGTCGTCT-3’
		5’-AGACGACGTTGTGCTTAGCTTGTTTGTATTCCTTGAAGAAGATGGTGCGCTCCTGGACG/iCy3dT/AGCCTTCGTTTCTGGTGCGT-biotin-3’
	LNA-modified DNA	5’-ACGCACCAGAAACGAAGGCTACGTCCAGGAGCGCACCATCTTCTTCAAGGAATACAAACAAGCTAAGCACAACGTCGTCT-3’
		5’-AGACGACGTTGTGCTTAGCTTGTTTGTATTCCTTGAAGAAGATGGTGCGCTCCTGGACG/iCy3dT/AGCCTTCGTTTCTGGTGCGT-biotin-3’

Figure S6	Internally labeled dsDNA	5'-ACGCACCAGAAACGAAGGCTACGTCCAGGAGCGCACCATCTTCTTCAAGGAATACAAACAAGCTAAGCACAACGTCGTCT-3'
		5'-AGACGACGTTGTGCTTAGCTTGTTTGTATTCCTTGAAGAAGATGG/iFAMdT/GCGCTCCTGGACGTAGCCTTCGTTTCTGGTGCGT-3'
Figure S12	Protospacer $16\text{-}20_{mm}$ DNA	5'-ACGCACCAGAAACGAAGGCTACGTCCAGGAGCGCGTACGCTTCTTCAAGGAATACAAACAAGCTAAGCACAACGTCGTCT-3'
		5'-AGACGACGTTGTGCTTAGCTTGTTTGTATTCCTTGAAGAAGCGTACGCGCTCCTGGACG/iCy3dT/AGCCTTCGTTTCTGGTGCGT-biotin-3'
Figure S5	80-bp dsDNA-2	5'-ACGCACCAGAAACGAAGGCTACGTCCAGGAGCGCTGGTAGAAGAAGTTCCTTATCAAACAAGCTAAGCACAACGTCGTCT-3'
		5'-AGACGACGTTGTGCTTAGCTTGTTTGATAAGGAACTTCTTCTACCAGCGCTCCTGGACGTAGCCTTCGTTTCTGGTGCGT-3'
	sgRNA	5'GGAUUCGUCGGUUCAGCGACGAUAAGCCGAGAAGUGCCAAUAAAACUGUUAAGUGGUUUGGUAACGCUCGGUAAGGUAGCCAAAAGGCUGAAACUCCGUGCACAAAGACCGCACGGACGCUUCACAUAUAGCUCAUAAACAAGGGUUUGCGAGCUAGCUUGUGGAGUGUGAACAUAAGGAACUUCUUCUACCA-3'
Figures 1-5, S2, S3, S5-S15	crRNA	5'-GUUUGCGAGCUAGCUUGUGGAGUGUGAACUAUUCCUUGAAGAAGAUGGU/Cy5/3'
	tracrRNA	5'GGAUUCGUCGGUUCAGCGACGAUAAGCCGAGAAGUGCCAAUAAAACUGUUAAGUGGUUUGGUAACGCUCGGUAAGGUAGCCAAAAGGCUGAAACUCCGUGCACAAAGACCGCACGGACGCUUCACAUAUAGCUCAUAAAC-3'
	sgRNA	5'GGAUUCGUCGGUUCAGCGACGAUAAGCCGAGAAGUGCCAAUAAAACUGUUAAGUGGUUUGGUAACGCUCGGUAAGGUAGCCAAAAGGCUGAAACUCCGUGCACAAAGACCGCACGGACGCUUCACAUAUAGCUCAUAAACAAGGGUUUGCGAGCUAGCUUGUGGAGUGUGAACUAUUCCUUGAAGAAGAUGGU-3'
Figure 2E-F	AsCas12f1 binding site	5'…TGGTTTAGTGAACCGTCAGATCCGCTAGCGCTAC…3'
		5'…GTAGCGCTAGCGGATCTGACGGTTCACTAAACCA…3'

The matched sequences between protospacer DNA and RNAs are colored cyan, and mismatched sequences are underlined. PAM sequences are colored orange. The LNA-modified region is highlighted with a dark gray rectangle. Unmatched double-stranded DNA is highlighted with a yellow rectangle.

Reference

S1. Sun, B. and Wang, M.D. (2017) Single-Molecule Optical-Trapping Techniques to Study Molecular Mechanisms of a Replisome. *Methods Enzymol*, 582, 55-84.
